# Noise performance of frequency modulation Kelvin force microscopy

**DOI:** 10.3762/bjnano.5.1

**Published:** 2014-01-02

**Authors:** Heinrich Diesinger, Dominique Deresmes, Thierry Mélin

**Affiliations:** 1 Institut d’Electronique, Microélectronique et Nanotechnologie (IEMN), CNRS UMR 8520, CS 60069, Avenue Poincaré, 59652 Villeneuve d’Ascq, France

**Keywords:** dynamic, frequency noise, Kelvin force microscopy, noise performance, phase noise, thermal excitation

## Abstract

Noise performance of a phase-locked loop (PLL) based frequency modulation Kelvin force microscope (FM-KFM) is assessed. Noise propagation is modeled step by step throughout the setup using both exact closed loop noise gains and an approximation known as “noise gain” from operational amplifier (OpAmp) design that offers the advantage of decoupling the noise performance study from considerations of stability and ideal loop response. The bandwidth can be chosen depending on how much noise is acceptable and it is shown that stability is not an issue up to a limit that will be discussed. With thermal and detector noise as the only sources, both approaches yield PLL frequency noise expressions equal to the theoretical value for self-oscillating circuits and in agreement with measurement, demonstrating that the PLL components neither modify nor contribute noise. Kelvin output noise is then investigated by modeling the surrounding bias feedback loop. A design rule is proposed that allows choosing the AC modulation frequency for optimized sharing of the PLL bandwidth between Kelvin and topography loops. A crossover criterion determines as a function of bandwidth, temperature and probe parameters whether thermal or detector noise is the dominating noise source. Probe merit factors for both cases are then established, suggesting how to tackle noise performance by probe design. Typical merit factors of common probe types are compared. This comprehensive study is an encouraging step toward a more integral performance assessment and a remedy against focusing on single aspects and optimizing around randomly chosen key values.

## Introduction

Surface potential imaging in combination with atomic force microscopy in ultrahigh vacuum is based on the measurement of electrostatic forces in amplitude modulation Kelvin force microscopy (AM-KFM) [1] or the measurement of the electrostatic force gradient in FM-KFM [2], in analogy with the FM mode used in noncontact atomic force microscopy (nc-AFM) [3]. The FM-KFM mode is often favored either because when a higher derivative of the probe–sample capacity is used, it is expected to be more sensitive to the very extremity of the tip [4], or because the use of probes with an increased fundamental resonance frequency makes the use of higher harmonics for simultaneous surface potential imaging inaccessible to the bandwidth of the deflection detector.

Previous studies of noise propagation often retrieve the general expression of frequency noise of a thermally excited harmonic oscillator and are not specific to a PLL based setup, and furthermore, do not extend to the noise in the KFM signal. The pioneer work on nc-AFM, [3] already mentions frequency noise for the first time in the context of nc-AFM, but takes into account only thermal probe excitation noise. Fukuma et al. [5] performed a detailed study on optimizing the probe deflection sensor and compare the measured noise power spectral density (PSD) at the PLL frequency output to the theoretical values derived from both thermal probe excitation and deflection sensor noise. Kobayashi et al. [6] focus on noise propagation in low quality factor (low-*Q*) environments for the application in liquids. Polesel-Maris et al. [7] studied the noise propagation in both amplitude and phase feedback loops of a nc-AFM as a function of the feedback controller settings, and showed that at a weak probe–surface interaction, the feedback loops can be considered independently whereas at a strong interaction, they become coupled. In our work on the dynamic behavior of AM-KFM [8], we studied the noise propagation from sensor displacement noise to the Kelvin voltage output. Giessibl et al. [9] compared qPlus and length-extension resonator (LER) sensors with respect to four noise sources: thermal excitation, sensor displacement noise, oscillator noise and thermal drift noise. The impact of all noise sources on frequency noise was discussed. Finally, Lubbe et al [10] numerically modeled noise propagation from sensor displacement noise to frequency noise of a PLL based nc-AFM depending on filter settings.

In this work, the noise propagation of a PLL based FM-KFM is studied by measuring and analytically modeling noise at different stages of the setup starting from the beam deflection signal, via the phase detector and the PLL outputs up to the Kelvin output voltage. The concept of noise gain allows for decoupling noise performance from the optimization of bandwidth and stability. It is commonly used in designing operational amplifier circuits. The noise PSD is modeled as if the bandwidth was unlimited and later, the bandwidth is chosen as a function of the acceptable signal fluctuation. This approach is appropriate because (1) increasing the closed loop bandwidth of a stable feedback loop above a certain frequency does not alter the noise PSD shape at the onset up to that frequency, and (2) stability and bandwidth are in many cases, including the described setup, not the bottleneck, i.e., constant gain can easily be achieved up to a frequency above the one at which the total output noise exceeds an acceptable value. The modeled noise PSD is in agreement with the measured one, showing that no significant noise contribution is added by the PLL. Since in FM-KFM the frequency shift signal is shared by both distance and potential control loops, a design rule for choosing the AC modulation frequency is proposed that ensures making best use of the available PLL bandwidth with negligible crosstalk between the loops and that yields equal bandwidth for both loops. The Kelvin output noise reduces to a compact analytic expression in terms of probe merit factors and a criterion for the transition between dominating detector and thermal excitation noise is derived. Noise optimization can then be approached via probe design after identifying the bottlenecks and addressing the respective parameters. The work is an approach toward a more integral view of KFM performance. A limit to optimization is the complicated interdependence of probe and detector parameters that for a practical implementation prevent reaching the ultimate theoretical limit imposed by the uncertainty principle.

## Gain and noise gain

**Figure 1:**
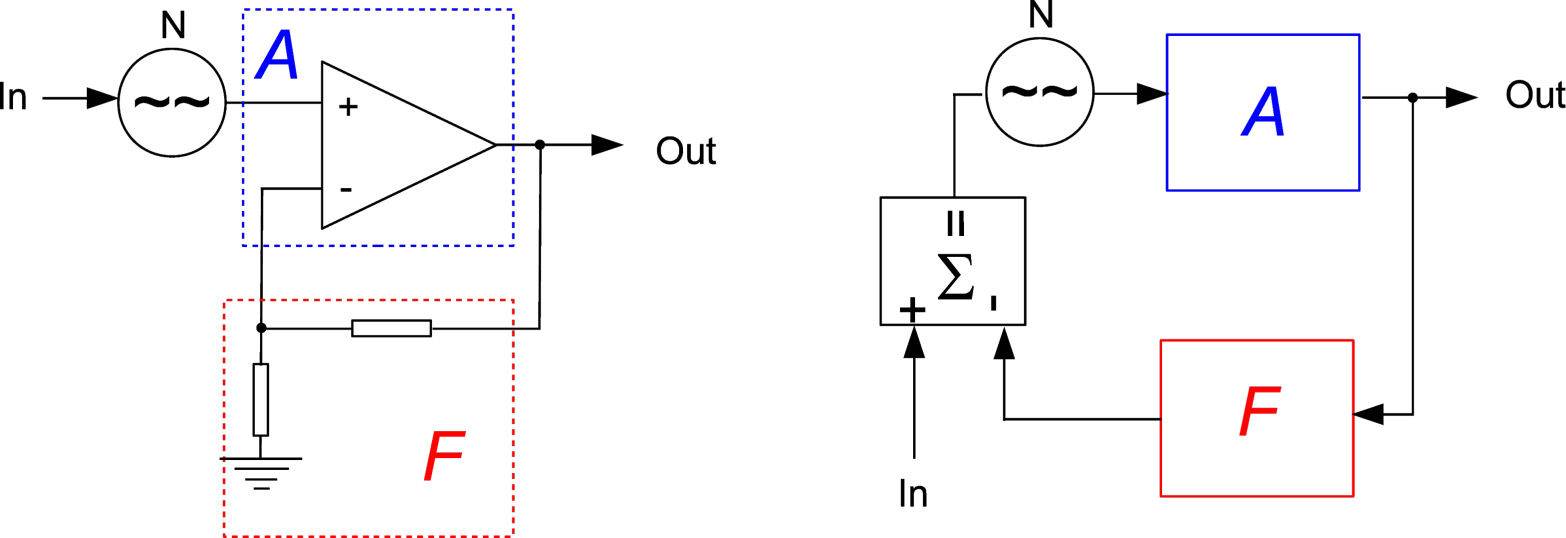
An OpAmp circuit and its equivalent circuit of forward gain *A* and feedback gain *F*.

For studying the noise propagation across the control loops, the concept of noise gain from OpAmp circuits is adopted. Figure 1 shows an OpAmp in a typical configuration and its decomposition into forward and feedback gain, adder and noise source. Later on, each of the KFM control loops will be represented by a similar equivalent circuit. Generally, the feedback gain *F* corresponds to a PI (proportional, integral) controller.

The output signal Out is written as function of the input signal In, the noise *A*_n_ and the gains:

[1]
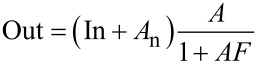


In this case the signal gain is equivalent to the noise gain

[2]
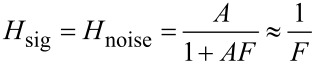


Depending on where the noise generator is inserted in the loop, the gains for signal and noise can be different as will be shown later. The approximation, although valid only in the operating bandwidth below the closed loop cutoff frequency, is widely accepted as the noise gain. The reason will be explained later.

## The PLL controller

**Figure 2:**
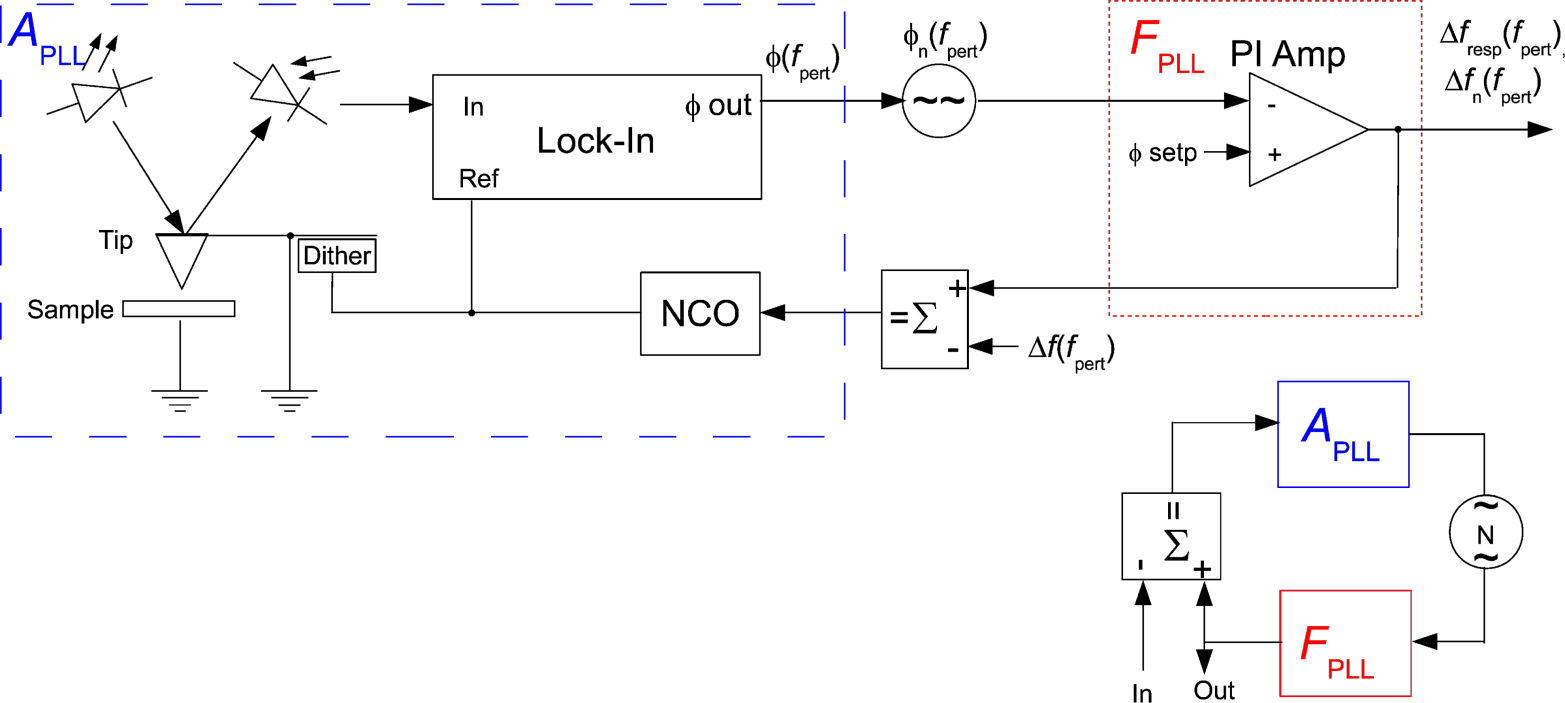
PLL: in the blue box, the components belonging to the forward gain *A*_PLL_, i.e., NCO, probe, optical beam detection, and lock-in amplifier used as phase detector, and in the red box, the PI controller representing the feedback gain *F*_PLL_, comparable to Figure 1. At the lower right, the equivalent circuit similar to Figure 1.

Figure 2 shows the setup of the PLL and the attribution of its components to the blocks *A* and *F* similar to Figure 1. The input is the resonance frequency variation Δ*f* of the tip, which is subject to external influence (van-der-Waals or electrostatic tip–sample interaction), and which is to be tracked by a numerically controlled oscillator (NCO) that drives the piezo dither. To match the oscillator to the resonance frequency of the tip, the deflection of the tip is detected, and the phase shift with respect to the drive signal is determined by a lock-in amplifier. The phase shift is compared to a setpoint, and the error signal is amplified by a PI controller that controls the NCO with the objective of keeping the drive frequency matched to the resonance frequency. A perturbation can be injected to an input of a signal adder (as indicated) to study the loop response, or by modulating the resonance frequency of the probe, e.g., by exposing it to an electric field, which shall both yield the same closed loop response.

### Phase detector gain - phase as function of frequency shift

We shall study the phase difference between a passive oscillator and a frequency modulated drive signal. If a resonator described by a quality factor *Q* and a resonance frequency *f*_0_ is excited by a frequency modulated drive force with an excursion *f*_exc_ and a modulation frequency *f*_pert_:

[3]
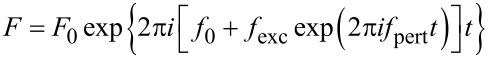


The phase shift is, without any assumptions about frequency excursion, width of the resonance peak, or modulation frequency exactly:

[4]
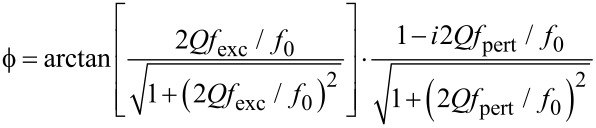


This can be derived heuristically by knowing that the phase is the integral over frequency difference in the regime of high modulation frequency *f*_pert_, but that the phase shift is capped by the extrema of the arctan function in the regime of steady excitation since one oscillator is passive. The same result had been found by Portes et al. [11] by solving the differential oscillation equation. This general equation yields the approximations for particular cases below that are so frequently found in the literature. It is noteworthy that the phase is generally complex, i.e., the phase difference is itself dephased with respect to the frequency modulation at *f*_pert_.

For this result, it is irrelevant whether the frequency difference is the result of applying a perturbation at the entrance of the NCO or of detuning the cantilever frequency. Since our digital AFM controller does not provide the option of modulating the excitation frequency, we will study the PLL response by perturbing the resonance frequency of the tip by applying a voltage between tip and sample. The first task is to determine the frequency shift induced as a function of the voltage and the fixed tip–sample distance of some tens of nanometers for the static case *f*_pert_ = 0.

**Figure 3:**
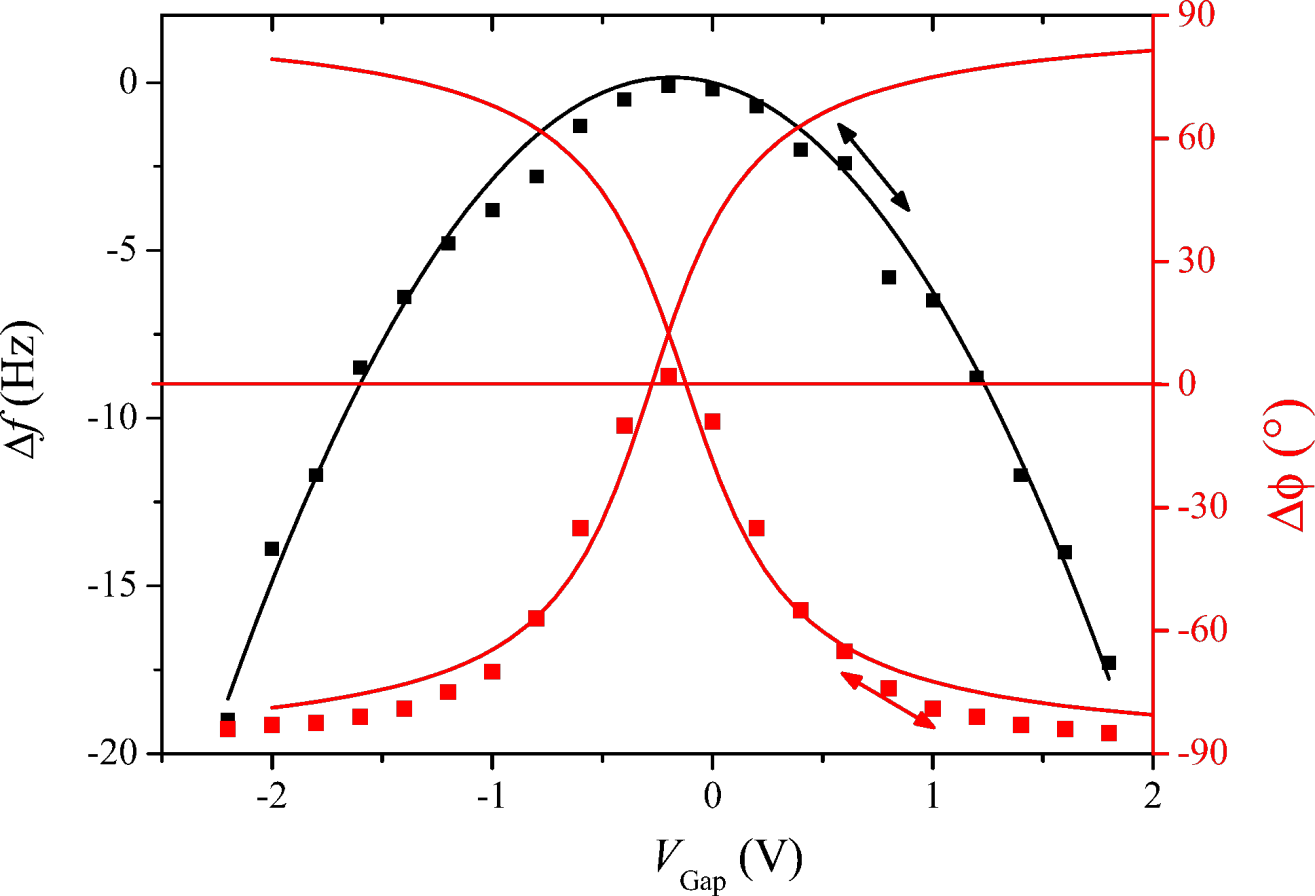
Resonance frequency shift resulting from applying a voltage between a retracted tip and sample (black, left scale) and phase shift resulting of exciting at constant frequency (red, right scale). The arrows indicate AC and DC bias applied in the dynamic study of the phase modulation, leading to Figure 4.

Figure 3 shows the frequency shift Δ*f* as a function of the voltage, measured by acquiring a resonance curve per voltage value (black squares and black solid line parabola fit). It also shows the static phase shift under excitation at constant frequency *f*_0_ (red squares), which is then shifted from the actual resonance by Δ*f* due to the influence of the electric field. Then, Equation 4 reduces to

[5]
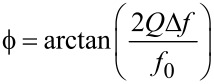


The red solid line is an arctan fit according to Equation 5. Note that the two branches of arctan functions do not intersect exactly at zero phase. This occurs if the resonance frequency of the tip drifts above the excitation frequency during the measurement. Consequently, the possible phase excursion may be higher than 90°.

**Figure 4:**
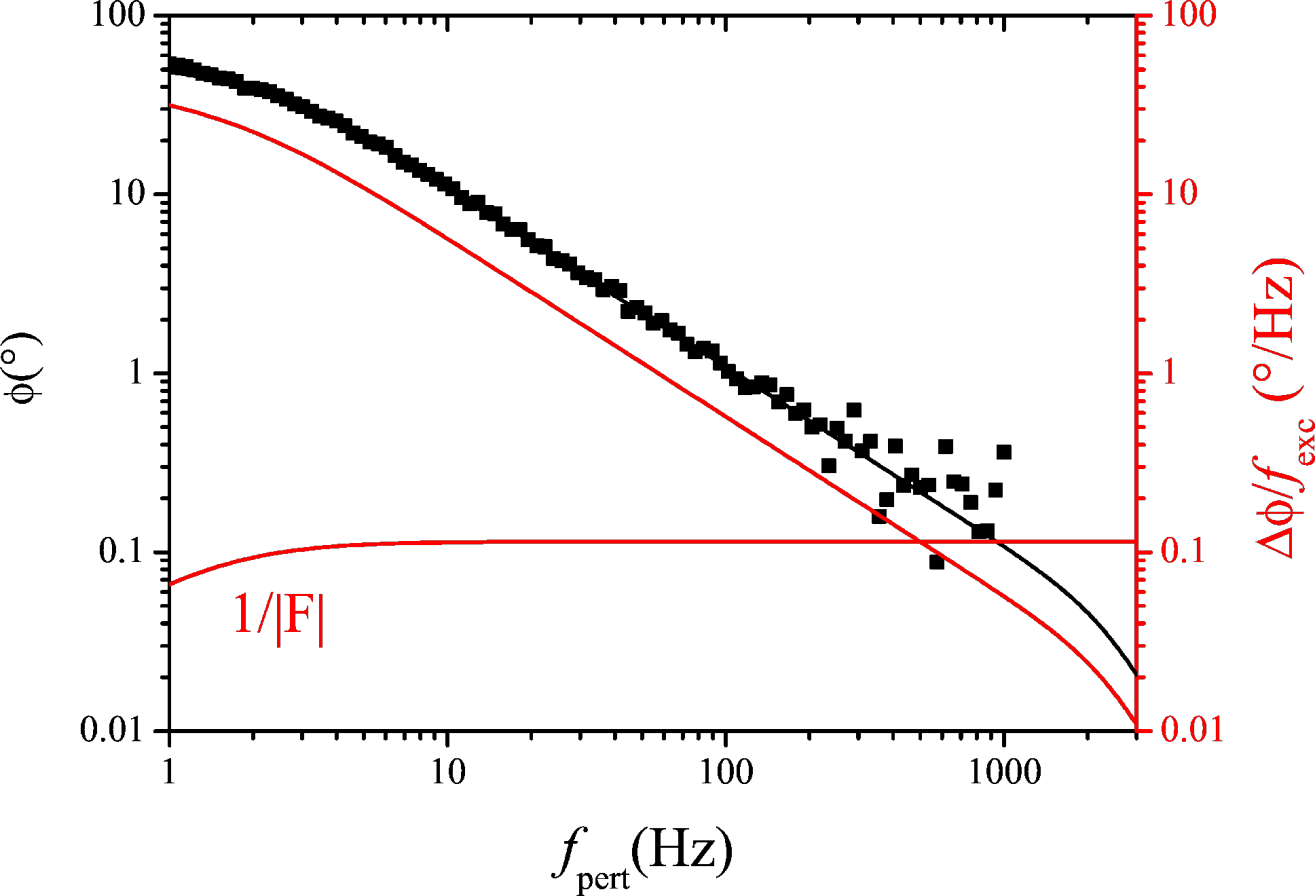
Phase detector output as function of modulation frequency (black squares), fitted with Equation 4, using *f*_exc_ = 1.9 Hz, multiplied by a Butterworth lowpass function with cutoff at 2.5 kHz. Also shown (red line), the gain *A*_PLL_ according to Equation 6, and the reciprocal feedback gain 1/*F*_PLL_ according to Equation 7.

Next, the forward response of the PLL, *A*_PLL_, is studied dynamically. This experiment has to be performed by applying a frequency modulation indirectly since the integrated lock-in module does not allow transfer function measurements by introducing a Δ*f* perturbation. For doing so, the tip is excited at constant frequency *f*_0_ = 61.835 kHz. Then, the resonance frequency is modulated by applying a bias containing both a DC and a smaller AC component of 0.8 V and 0.2 V respectively, and the phase detector output is recorded as function of modulation frequency of a small AC bias. We set the DC and the AC voltage components to aim at a frequency excursion of around *f*_exc_ ≈ 2 Hz as indicated by the arrow in Figure 3. The result is the spectrum shown in Figure 4 by black squares, giving the phase shift as a function of the modulation frequency.

It is fitted with Equation 4 multiplied by a lowpass function of the phase detector output filtering, a 2nd order Butterworth with *f*_c,LP_ = 2500 Hz cutoff frequency.

[8]
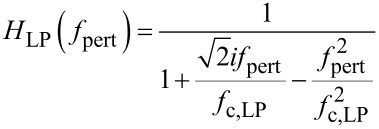


The best fit is obtained for an excursion of *f*_exc_ = 1.9 Hz and the previously found values for *f*_0_ = 61.835 kHz and *Q* = 22800 (see Experimental section).

For the following, a linear conversion gain of the phase detector must be defined in terms of phase divided by frequency excursion, as function of modulation frequency. Before we can divide Equation 4 by *f*_exc_, it is compulsory to approach the arctan function by its argument for small excursion, *f*_exc_ < *f*_0_/(2*Q*), since the definition of a gain implies a linear dependence. Then, Equation 4 simplifies and dividing by the excursion yields:

[6]
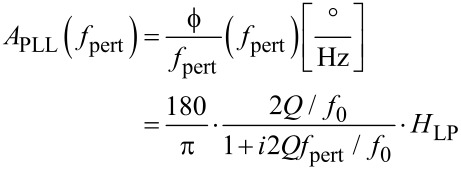


The approximation of the arctan function by its argument for small excursion is at the very limit of validity here because *f*_0_/(2*Q*) = 1.35 Hz and *f*_exc_ = 1.9 Hz. However when the phase detector is ulteriorly used in the closed PLL loop within its tracking bandwidth, the error is negligible: The closed loop gain is near unity in this range, meaning that the oscillator follows the (detuned) resonance frequency, and the frequency error remains at a fraction of the frequency excursion. The forward gain Equation 6 will be used to model both the closed loop PLL response and the shape of frequency noise PSD. It is also shown on Figure 4 as red curve. At the same time, the reciprocal value of the feedback gain *F*_PLL_ is displayed. The feedback circuit is a PI (proportional, integral) amplifier with the following response *F*_PLL_:

[7]
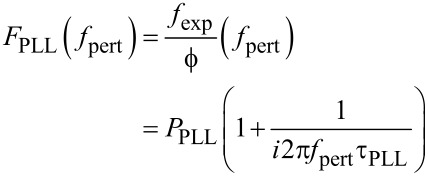


The controller software automatically sets the time constant of the phase locked loop PI amplifier equal to the time constant of the phase detector lowpass function, τ_PLL_ = 2*Q*/2π*f*_0_, and the *P* gain such that the crossing with the forward gain occurs at the chosen PLL bandwidth of 1 kHz.

The 2.5 kHz lowpass *H*_LP_ of the phase detector output is also a consequence of the choice of 1 kHz PLL bandwidth and automatically set by the controller. In this case, the feedback parameters were *P*_PLL_ = 8.73 Hz/° and τ_PLL_ = 112 ms.

### PLL noise

**Figure 5:**
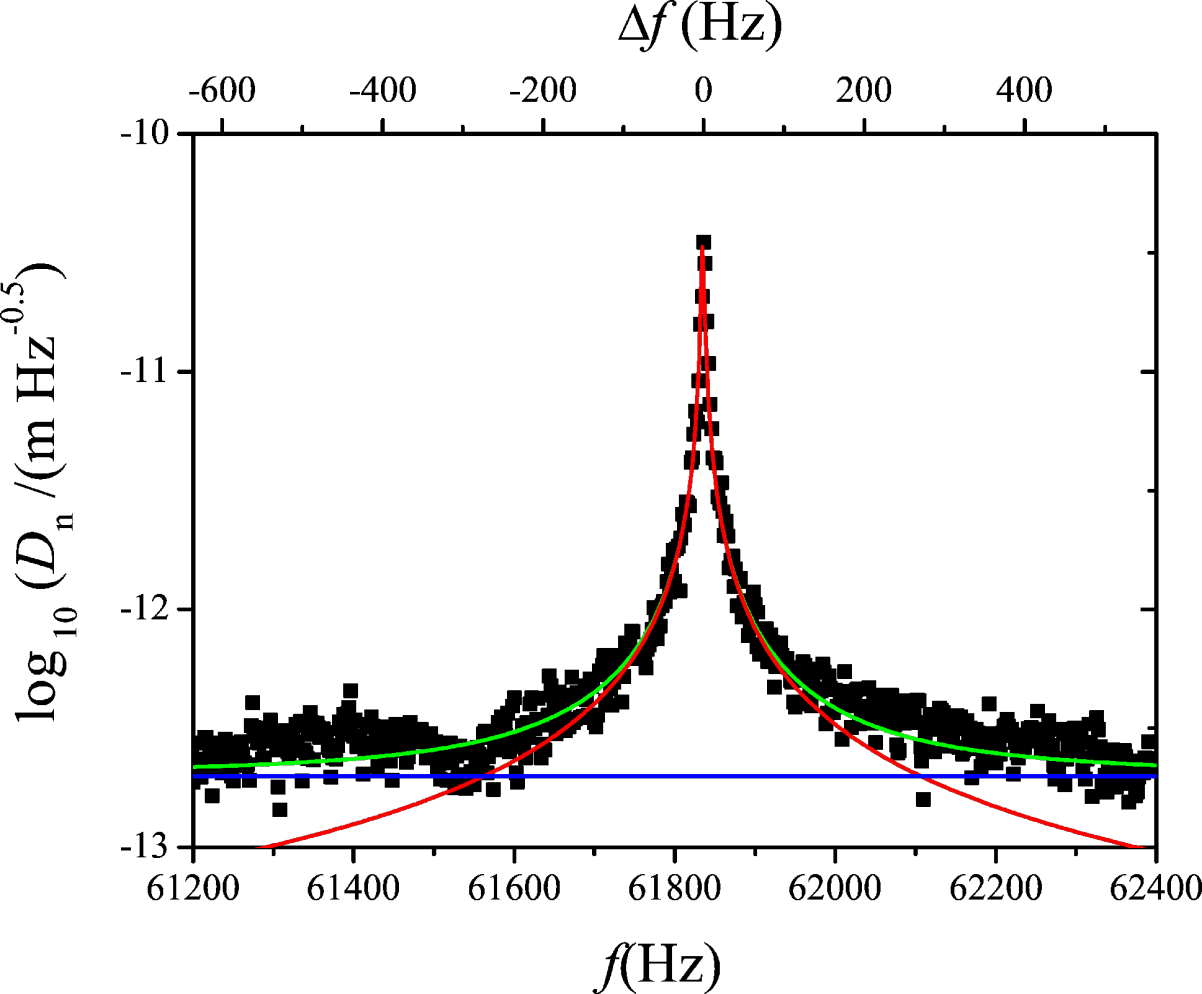
Noise PSD at the photodetector output. Fiteed with Equation 9 (green) and decomposition into thermal excitation noise (red) and constant detector noise *z*_n,S_ (blue).

First, a noise spectrum is measured at the output of the photodetector, without probe excitation. It is shown in Figure 5.

The deflection noise spectrum *D*_n_(*f*) contains a component due to thermal probe excitation plus a component due to detector output noise. The latter can be assumed to be constant over the relatively small frequency interval of the spectrum. The respective power spectral densities (PSD) in units of m/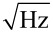 are uncorrelated and hence add in quadrature. The noise PSD *D*_n_(*f*) is therefore described by a quadrature sum of detector noise *z*_n,S_ and a Lorentzian component of the same *Q* and *f*_0_ as the resonance curve previously determined by the microscope controller:

[9]
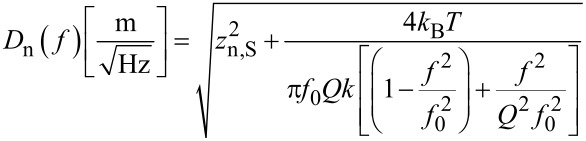


A curve fit with Equation 9 yields *k* = 1.2 N/m, *Q* = 22800, *f*_0_ = 61835 Hz and *z*_n,S_ = 2·10^−13^ m/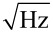.

The decomposition is also indicated in Figure 5. The optical beam deflection conversion gain leading to the scale was calibrated by using the method of reduced frequency shift [12] and was 0.15 nm/mV. The principle of this method is to maintain a constant reduced frequency shift by varying simultaneously the excitation amplitude and the frequency shift setpoint of the noncontact mode following a certain algorithm. Then, the lower turning point of the tip remains equidistant from the sample surface, and the motion of the z-piezo represents the shift of oscillation amplitude as response to varying excitation amplitude.

**Figure 6:**
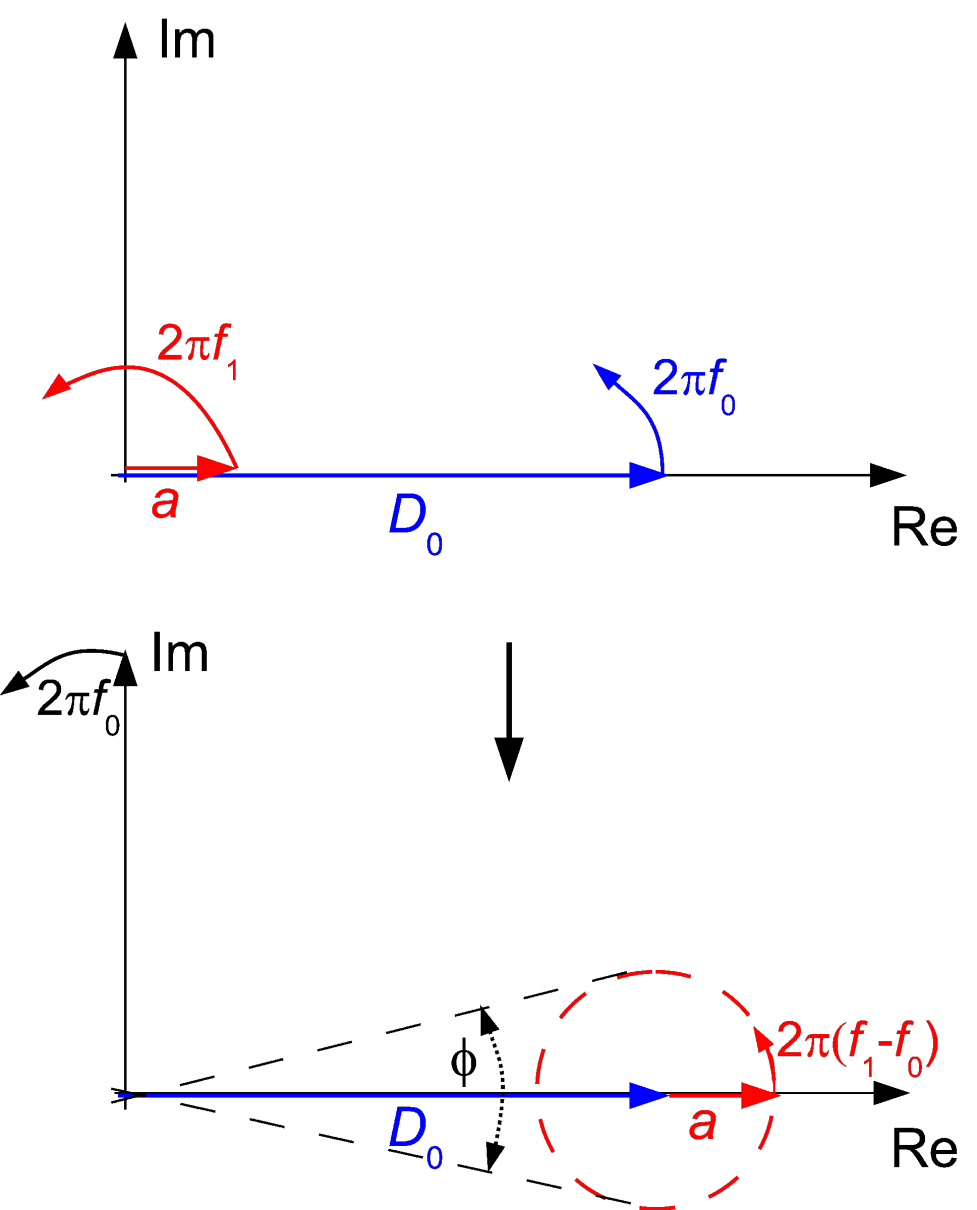
Vector diagram showing the impact of amplitude noise on phase noise in the complex plain: main vector and a small perturbation of amplitudes *D*_0_ and *a* and at frequencies *f*_0_ and *f*_1_ respectively (upper drawing) and their resulting sum in the reference plane rotating at *f*_0_ (lower drawing).

Next, the noise propagation throughout PLL and Kelvin loop are studied. In order to be able to model the noise by the approach of noise gains as in Figure 1, it is necessary to present it by a noise source inserted between blocks *A*_PLL_ and *F*_PLL_. We shall now calculate how the displacement noise at the photodetector output transforms into phase noise at the phase detector output, which is represented by the phase noise generator of Figure 2. Figure 6 shows the vector diagram in the complex plane of a signal *D*_0_cos(2π*f*_0_*t*), representing the tip deflection, plus a spurious small signal *a*cos(2π*f*_1_*t*) as a representation of the deflection detector noise. If demodulated by a lock-in amplifier at *f*_0_, in the reference system rotating at *f*_0_, the *D*_0_ vector is stationary and *a* is rotating around the end of *D*_0_ at *f*_1_ − *f*_0_.

The imaginary projection is then

[10]
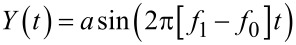


The phase is for *a* << *D*_0_

[11]
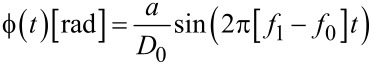


A small signal vector *a* at *f*_1_ = *f*_0_ + *f*_pert_ causes a phase oscillation at *f*_pert_. If *a* was rotating at *f*_1_ = *f*_0_ − *f*_pert_, it would also cause a phase oscillation at *f* but with opposite sign, and hence two vectors *a* at opposite difference frequencies would add arithmetically and cancel. Regarding the phase noise at a frequency *f*_pert_, the spurious superimposed oscillations are replaced by the respective noise densities at frequencies *D*_n_(*f*_0_ ± *f*_pert_)[V/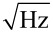]. Since the two noise components are uncorrelated, the densities add in quadrature:

[12]
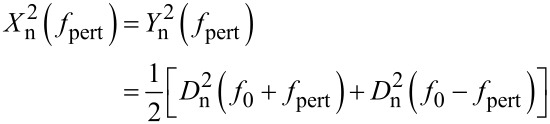


The factor 1/2 applies because half of the power spectral density (PSD) is projected onto each real and imaginary axis in the complex plane. In analogy with Equation 11, the phase noise PSD 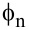, becomes:

[13]
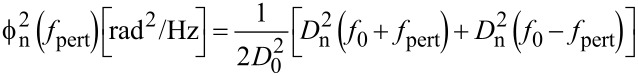


Due to the symmetry of the Lorentzian with its high quality factor, it is sufficient to use one branch of the Lorentzian, and expressed in degrees we obtain:

[14]
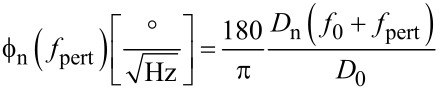


This expression gives the phase noise with the use of a lock-in amplifier. It had been derived in a similar way by Rast et al. [13]. It may not be valid for other phase comparators, e.g., edge triggered ones. It is basically a translation by *f*_0_ of the deflection noise PSD. The translation of the Lorentzian component of the deflection noise yields a first order lowpass with respect to *f*_pert_ with a cutoff frequency *f*_0_/(2*Q*), whereas the constant detector shot noise *z*_n,S_ is invariant under translation, yielding for the total phase noise the quadrature sum:

[15]
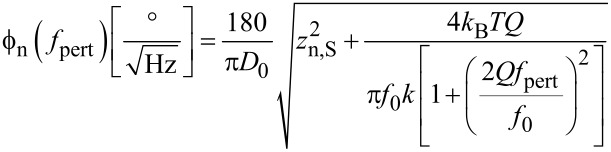


**Figure 7:**
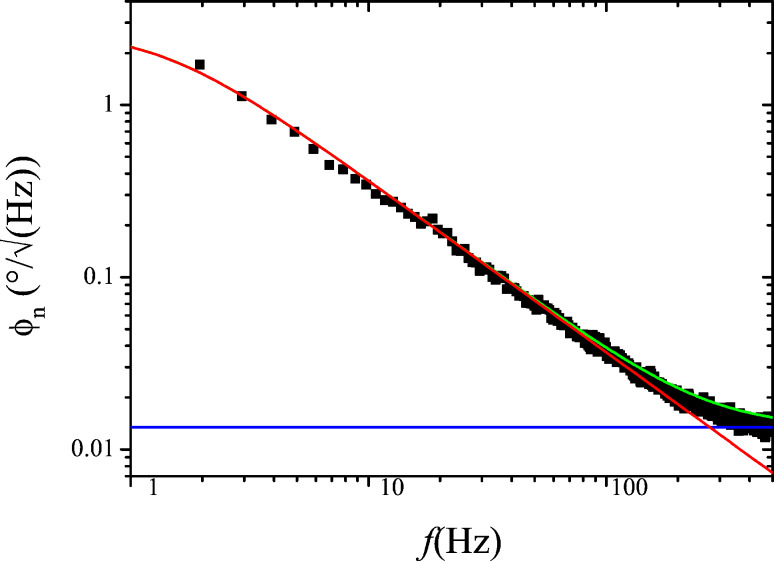
Phase noise PSD at the lock-in phase detector output in open PLL loop and under probe excitation at *D*_0_ = 0.75 nm: measured (black squares), fitted according to Equation 15 (green), and decomposed into detector noise (blue, constant) and thermal excitation contribution (red, lowpass) according to the two terms of Equation 15.

Figure 7 shows the measured phase noise at the phase detector output in open PLL, and in green the fit according to Equation 15. The parameters *z*_n,S_, *f*_0_, *Q* and *k* were kept identical to the ones of the curve fit of Figure 5, whereas the oscillation amplitude had to be adjusted to *D*_0_ = 0.85 nm. The red and blue lines show the fit decomposed into thermal excitation and detector noise components, respectively.

### PLL closed loop gain

With the known transfer functions *A*_PLL_ from Equation 6 and *F* from Equation 7, the closed loop response of the PLL can be computed. For the equivalent circuit of Figure 2, we find a signal gain

**Figure 8:**
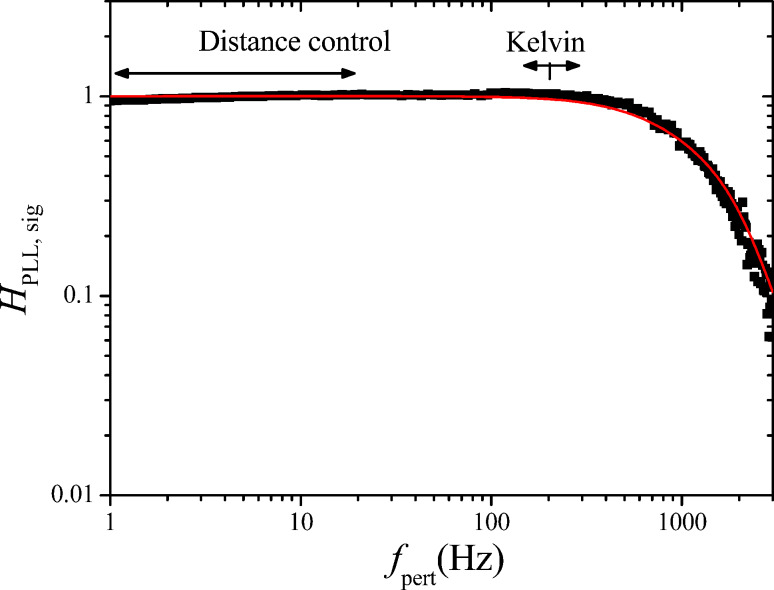
Closed loop PLL response: measured (black squares) and computed (red line) according to Equation 16.

[16]
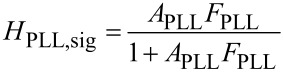


which is plotted along with the measured response in Figure 8. The computation is performed on complex transfer functions and the plot only shows the modulus of the result. Note that in Equation 16, *A*_PLL_ and *F*_PLL_ are complex and only in the end of the computation, the module is calculated.

### PLL closed loop noise

With the known forward and feedback gains of the PLL loop, the closed loop noise output spectrum *f*_n_ of the PLL is modeled. Since the noise source of Figure 2 is located differently between blocks *A* and *F* than that of Figure 1, the noise gain is different from the signal gain in contrast to the operational amplifier example, and writes

[17]
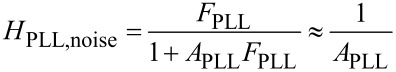


The frequency noise PSD *f*_n_ at the output of the PLL is

[18]
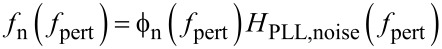


The PLL phase detector output noise PSD modeled by Equation 15 is used as 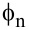 input. The calculation of the noise gain is also performed on complex gains.

**Figure 9:**
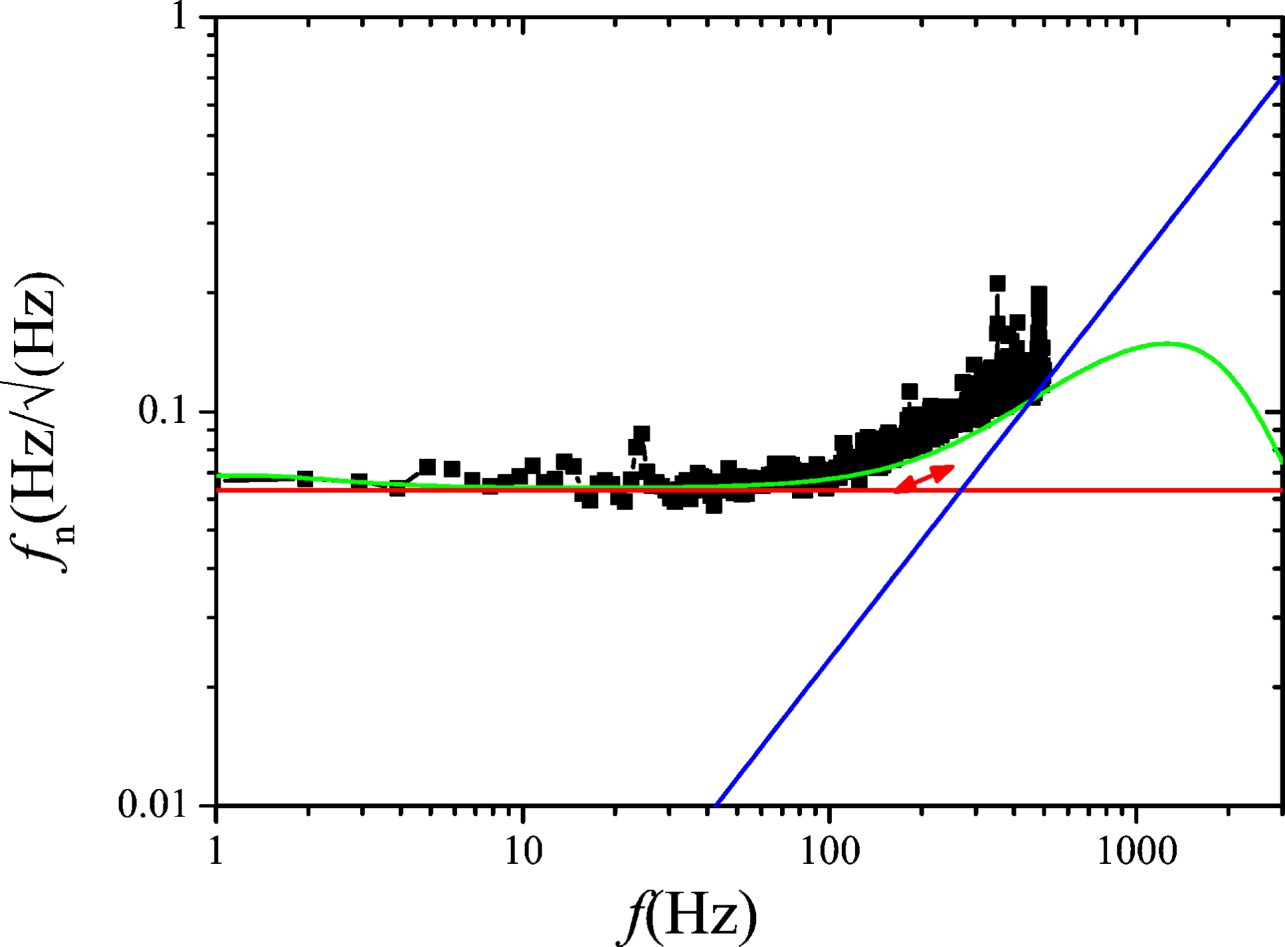
Output noise PSD of the PLL in closed loop configuration: measured (black squares) and modeled according to Equation 18 (green) from the previously determined gains and phase noise spectrum. The decomposition into thermal excitation contribution (red, constant) and detector noise (blue, rising) are also shown; the decomposed spectra were modeled with simplified noise gains *H*_noise,PLL_ ≈ 1/|*A*_PLL_|.

Figure 9 shows the PLL closed loop PSD of noise *f*_n_, (black), up to 500 Hz, the limit of the integrated spectral analyzer, and the numerically computed noise PSD (green) obtained from phase noise and gains *A*_PLL_ and *F*_PLL_. Furthermore, the noise PSD decomposed into thermal noise (red) and sensor noise (blue) is computed according to the approximation of Equation 17 (see Equation 19 below).

Regarding the approximation of Equation 17, we note that it is valid in the range in which the closed loop gain is unity and |*A*_PLL_*F*_PLL_| >> 1. Hence, the approximation does not predict the roll-off of the noise PSD beyond the closed loop bandwidth. Instead, it predicts infinite rise of the noise following the blue line of Figure 9. To use the approximation 1/*A*_PLL_, denoted “noise gain”, rather than the exact computation is the idea of the noise gain formalism and is justified by the following arguments:

- The roll-off of the noise PSD cannot be exploited anyway: if the loop is used beyond its cutoff frequency, it attenuates the signal as much as the noise. The image acquisition circuitry that samples data into pixels has an anti-aliasing low-pass filter with a cut-off frequency of half the sampling rate. There is no interest of using a loop bandwidth below that cut-off frequency since the response of the loop would then smooth the image at the same rate as it would smooth out noise.
- If the loop is inserted into a surrounding loop, then the closed loop gain of the former becomes the forward gain of the latter; consequently, the roll-off has two effects that compensate each other: first, it cuts off the noise PSD, but second, since the reciprocal of the inner loop closed loop gain becomes itself the (approximate) noise gain of the surrounding loop, it would amplify the noise PSD by as much as it had been attenuated before. Therefore, it is convenient to neglect the cutoff in noise propagation.
- Last, it is noteworthy that the closed loop cutoff frequency has no influence on the noise PSD at the onset below that cutoff frequency, e.g., the noise PSD from zero to 300 Hz is the same irrespective of whether the closed loop cutoff frequency is 500 Hz or 1 kHz. Therefore, it is convenient to first calculate the noise PSD as if the closed loop bandwidth was infinite, to determine over which frequency range the noise PSD can be integrated without exceeding an acceptable total signal fluctuation, and to limit bandwidth and sampling rate a posteriori. It will be discussed later to what extent the approach is feasible and whether stability issues can become the bottleneck.

Therefore among engineers the noise gain formalism is widely used but to our knowledge has not yet been applied to noise propagation in scanning probe microscopy. The PLL output noise PSD is now obtained using the noise gain formalism:

[20]
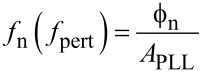


Regarding the PLL forward gain *A*_PLL_, Equation 6, it is noteworthy that the open loop gain of the phase as function of frequency excursion has the same frequency dependence as the thermal contribution of the phase noise, second term of Equation 15, i.e., a first order lowpass with cutoff frequency *f*_0_/(2*Q*). The quotient Equation 20 yields, when inserting the phase noise PSD from Equation 15 and PLL forward gain *A*_PLL_ from Equation 6:

[19]
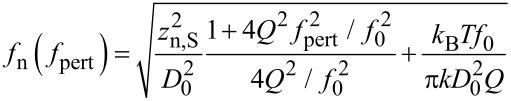


Hence, the thermal part of the frequency noise, is exactly constant over a range from zero to infinite frequency, which is the third term. It was derived by theorists and resumed by Giessibl and Kobayashi that a thermally excited harmonic oscillator is expected to have constant frequency noise PSD [5,14]. Here, we have provided the comprehensive step-by-step evidence for an experimental PLL setup with a driven passive resonator, yielding the same result. Controversial debate about the frequency noise of comparable PLL setups is still ongoing [15–16].

The sensor noise contribution is split into two contributions, the first term without frequency dependence, the second rising with *f*_pert_ above *f*_0_/(2*Q*), meaning above 1.35 Hz in our case, below which it has a plateau. We state that Equation 19 is identical to Equation 18 of reference [6], up to a factor of 2 in front of the sensor noise contributions. The first frequency independent term of our sensor noise has been referred to as “oscillator noise” by Kobayashi which was later also adopted by Giessibl [9]. Following our approach, it is arising merely from propagation of sensor noise throughout the PLL. This frequency-independent component of sensor noise is only found in modeling if the PLL forward gain, Equation 6, is derived exactly with the *f*_0_/(2*Q*) corner frequency, rather than an approximate 1/*f*_pert_ behavior. It is generally negligible in high-*Q* environments as in our setup. The good agreement between computed noise and experiment shows that here the frequency noise can be attributed solely to thermal excitation and sensor noise.

## The Kelvin loop

**Figure 10:**
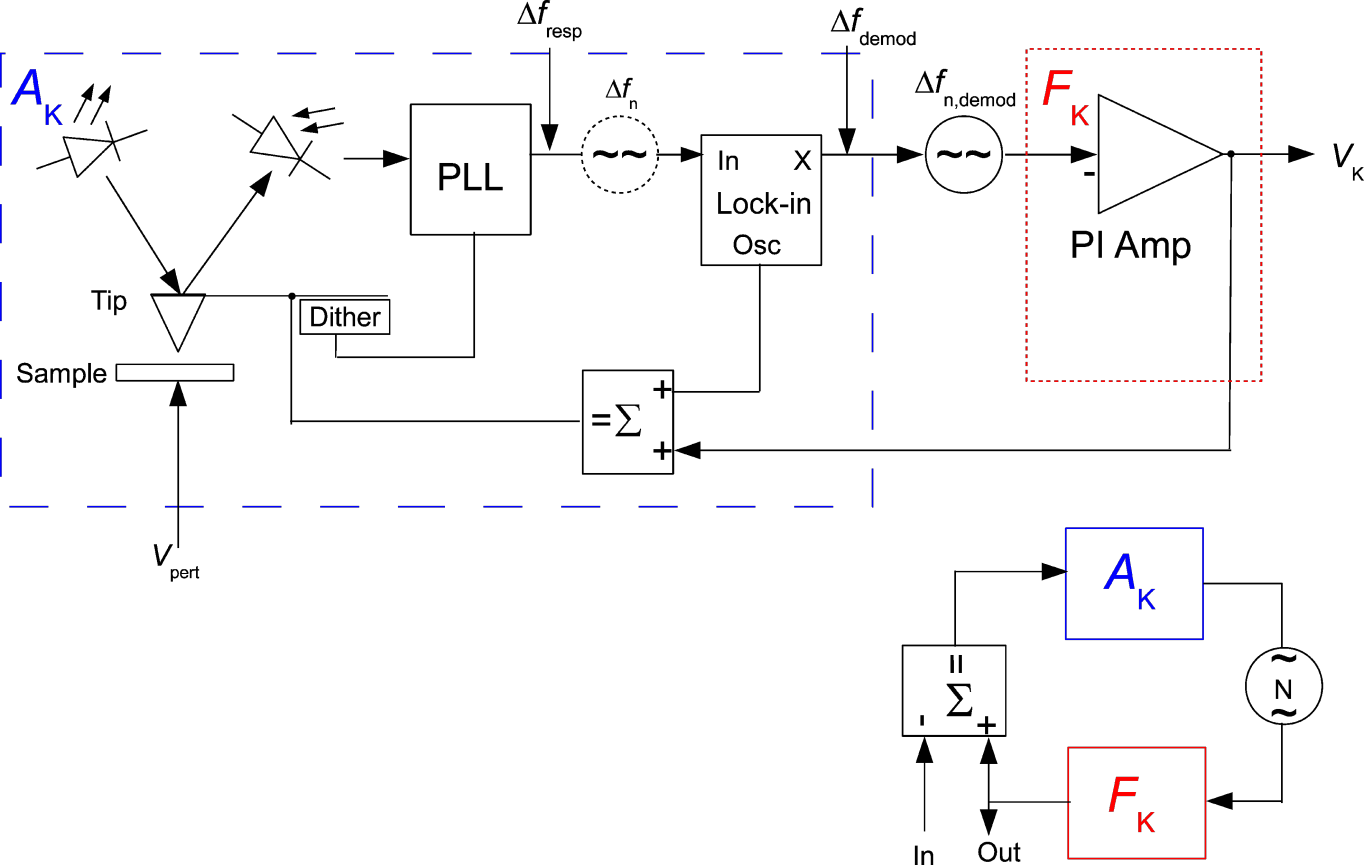
Kelvin loop and its equivalent circuit: the forward gain *A*_K_ is the transfer function between *V*_pert_ and (Δ*f*)_demod_ and contains the previously studied closed loop PLL, a Kelvin lock-in amplifier, and a signal adder (components in the blue box). The feedback gain *F*_K_ is a PI amplifier (red) that adjusts the tip voltage *V*_K_ to compensate the CPD. The output noise of the PLL controller is projected onto the *X* output of the Kelvin lock-in amplifier to facilitate the representation in an equivalent feedback circuit (lower right) for the calculation of noise in the Kelvin signal.

Figure 10 shows the setup of the Kelvin loop. The closed PLL loop of the previous section now presents a small part of the forward gain. The *A*_K_ block further contains a lock-in amplifier working at a frequency lower than the bandwidth of the PLL that modulates the gap voltage. It superposes an AC signal *V*_mod_ at a frequency within the operating range of the PLL and detects the resonance frequency modulation of the tip at this frequency. The output of block *A*_K_ is the demodulated frequency shift Δ*f*_demod_, while the input is the *V*_K_ component of the tip bias. The tip voltage superposition has two purposes: first to extract the polarity information of the gap voltage mismatch, and second to share the PLL bandwidth between Kelvin and distance controller: van-der-Waals and electrostatic interaction both shift the resonance frequency. A modulation of Δ*f* at a frequency within the bandwidth of the distance controller would cause the distance controller to retract the tip periodically. Therefore, by modulation and demodulation, the electrostatically induced tip frequency modulation is translated in a range above the cutoff frequency of the distance controller, but below the cutoff frequency of the PLL.

The contact potential difference (CPD) between tip and sample is indicated by a voltage applied to the sample. It may be due to a work function difference between the sample and the tip or due to a sample to which a bias is applied. The objective is to cancel the CPD by applying a Kelvin voltage to the tip such that CPD − *V*_K_ = 0.

### Open loop forward gain

The CPD, the Kelvin voltage, *V*_K_, and the AC voltage, *V*_mod_, cause an electrostatic field gradient that alters the resonance frequency:

[21]
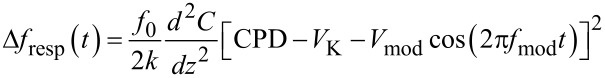


The assumption of a constant *d*^2^*C*/*dz*^2^ is an approximation for oscillation amplitudes smaller than the mean tip–sample distance. However, the voltage dependence is valid even if this condition is not exactly met. By expanding the square bracket of Equation 21, one gets

[22]
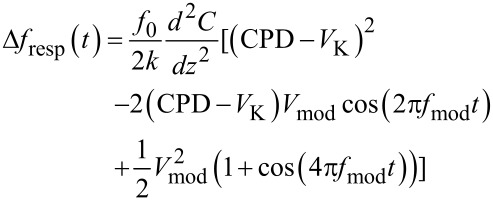


The term of interest is the mixed term at *f*_mod_ since it contains amplitude and sign of the CPD. It is detected by demodulating at *f*_mod_. The PLL response *H*_PLL_(*f*_mod_) applies before the demodulation. The static forward gain *A*_K,DC_ is:

[23]
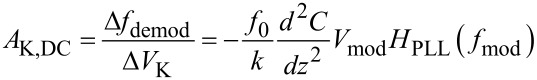


**Figure 11:**
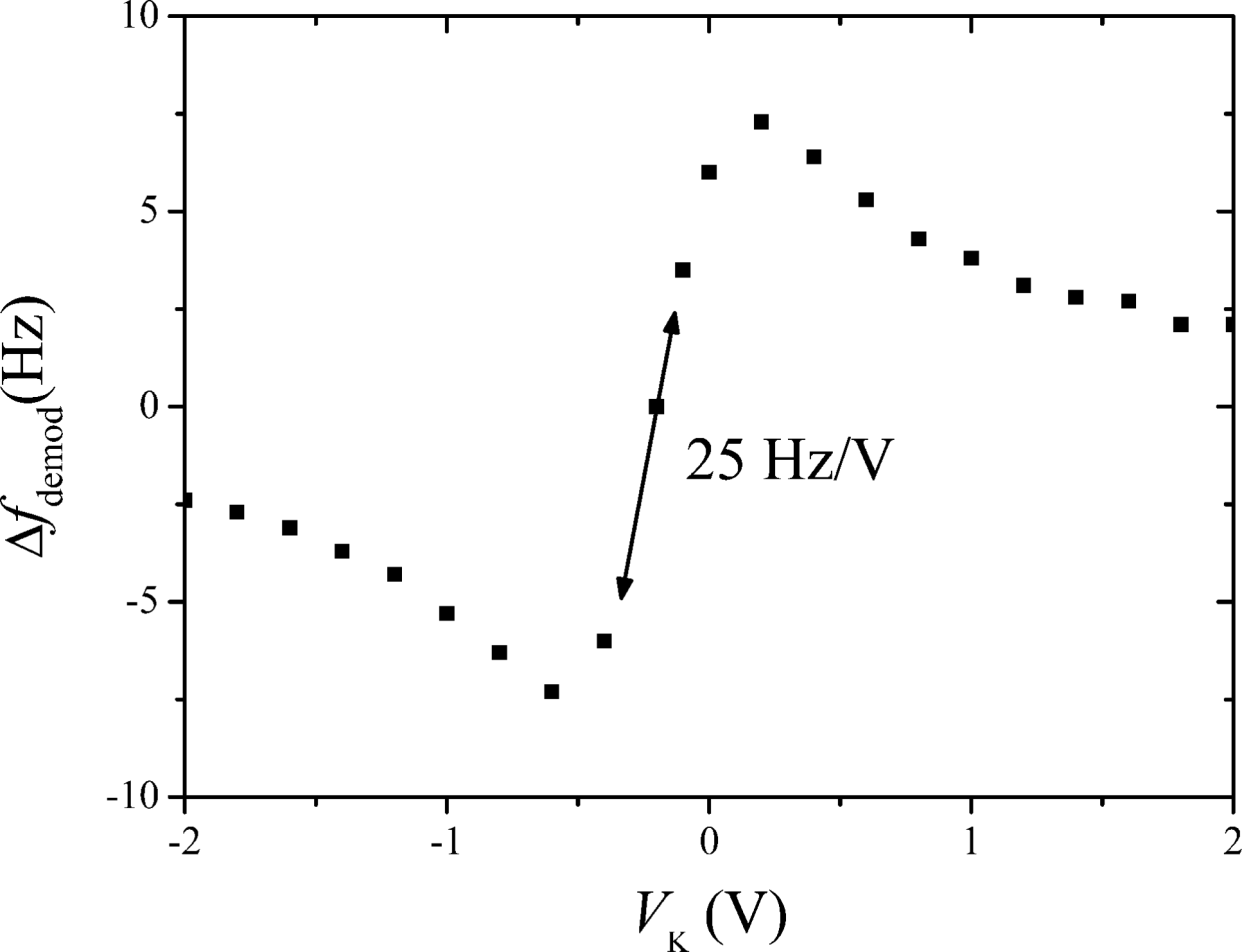
Measurement of static forward gain of the open Kelvin loop.

The static forward gain is determined with engaged distance control loop while using a setpoint of Δ*f* = −5 Hz with *V*_mod_ = 300 mV, and *f*_mod_ = 200 Hz. The demodulated error signal is then measured as a function of Δ*V*_K_, shown in Figure 11. The gain is 25 Hz/V.

Next, the forward gain is studied dynamically. Therefore, the DC voltage mismatch CPD − *V_K_* is replaced by an AC voltage *V*_pert_cos(2π*f*_pert_*t*) and Equation 22 becomes

[24]
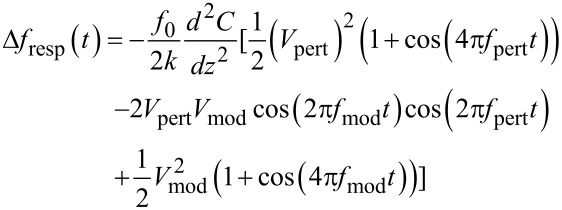


The mixed term will transform into two satellites at *f*_mod_ ± *f*_pert_ and Equation 23 becomes

[25]
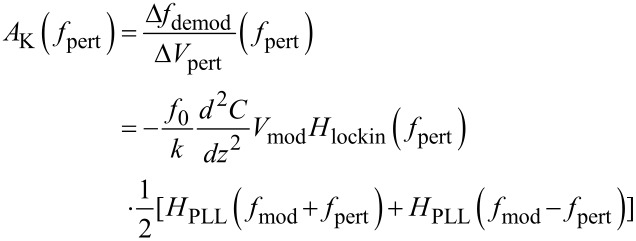


If the PLL response is flat and unity around *f*_mod_ ± *f*_pert_, above expression is equal to the static gain, multiplied by the output filtering of the Kelvin lock-in amplifier *H*_lockin_. The validity of Equation 25 requires that the distance control loop does not interfere with the Kelvin control loop. First, it must not modify, by tip–surface interaction, the PLL response, e.g., by modifying *Q* via dissipation; second, it must not respond periodically to the frequency modulations caused by the Kelvin loop. This means that *f*_mod_ − *f*_pert_ must be above the cutoff frequency of the distance control loop. The ranges of PLL bandwidth occupied by distance and Kelvin loop are indicated by the arrows in Figure 8. In a range of *f*_pert_ where the PLL closed loop has unity gain for *f*_mod_ ± *f*_pert_, Equation 25 can be approximated as

[26]
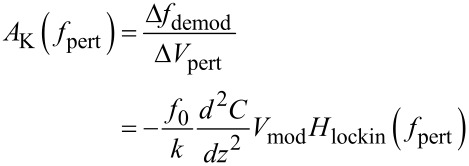


### Noise projection behind the Kelvin lock-in amplifier

Concerning the noise, if an equivalent control loop circuit in the sense of Figure 1 is to be applied, it is required to express the noise PSD at the interface of block *A* of Figure 10, meaning in the *X* output of the Kelvin lock-in amplifier. Hence the propagation of the noise PSD of the PLL output to the output of the Kelvin lock-in is now calculated. The projection of the PLL noise to the demodulated *X* output is the average between the satellites at *f*_mod_ ± *f*_pert_

[27]
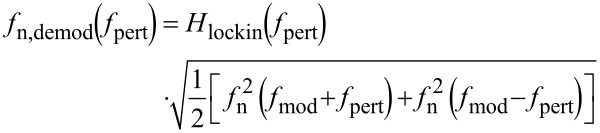


where *f*_n_ is given by Equation 19.

### Kelvin closed loop gain and noise

The loop is closed with a feedback gain *F*_K_(*f*_pert_) of a PI controller described by Equation 28:

[28]
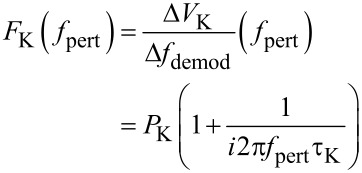


**Figure 12:**
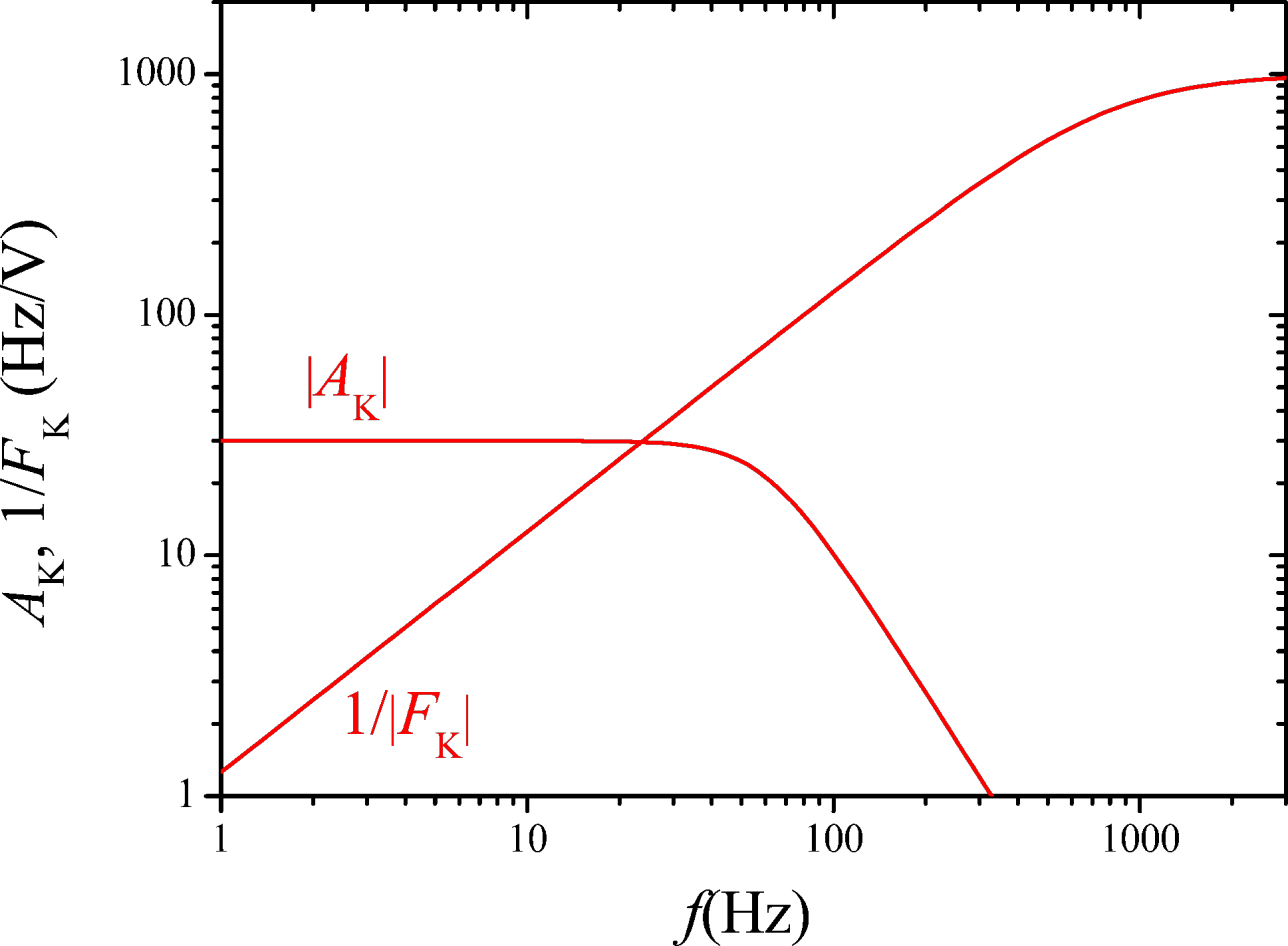
Schematic forward and reciprocal feedback response, for illustrating the choice of the Kelvin feedback parameters.

The feedback parameters are set to *P*_K_ = 1 mV/Hz and τ_K_ = 200 µs. To understand the choice of the parameters and their effect on the closed loop response, a schematics depicting both forward response |*A*_K_| and reciprocal of the feedback response, 1/|*F*_K_|, is shown in Figure 12.

The main point is that the 1/*F*_K_ responsecurve crosses, with its slope, the *A*_K_ responsecurve at a frequency where it is essentially constant. Many combinations of *P*_K_ and τ_K_ are possible that yield the same closed loop cut-off frequency because only the frequency of crossing matters, but not the height of the plateau of the |1/*F*_K_| function. In the *P*–*I* representation, the *I*_K_ component would need to be set to a specific value while the *P*_K_ could be varied in a wide range. With the known open loop forward gain and output noise PSD (Δ*f*)_n_ of the PLL, it is possible to calculate the closed Kelvin loop signal and noise gain according to Figure 10 to compare them to the measured spectra:

[29]
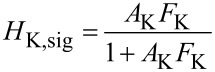


[30]
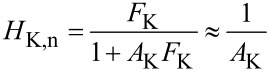


[31]
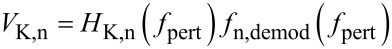


**Figure 13:**
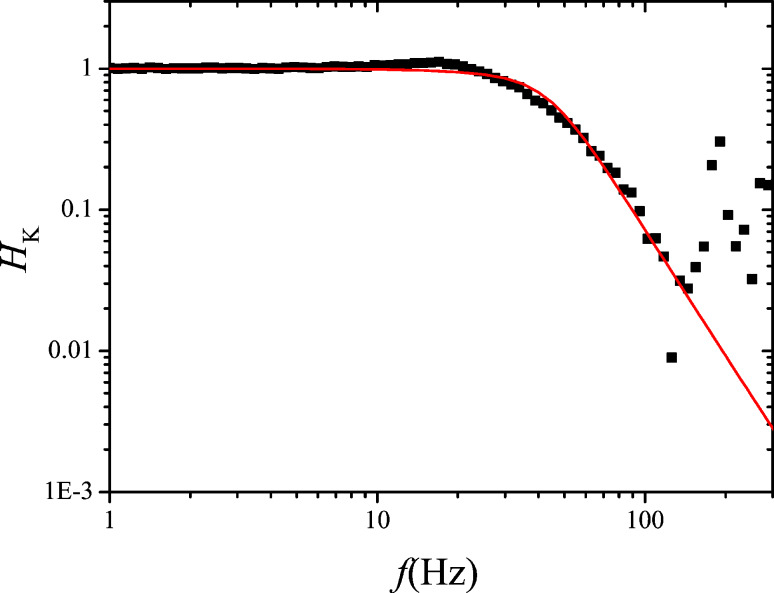
Measured (black squares) and calculated (red line) Kelvin closed loop gain of the setup of Figure 10.

**Figure 14:**
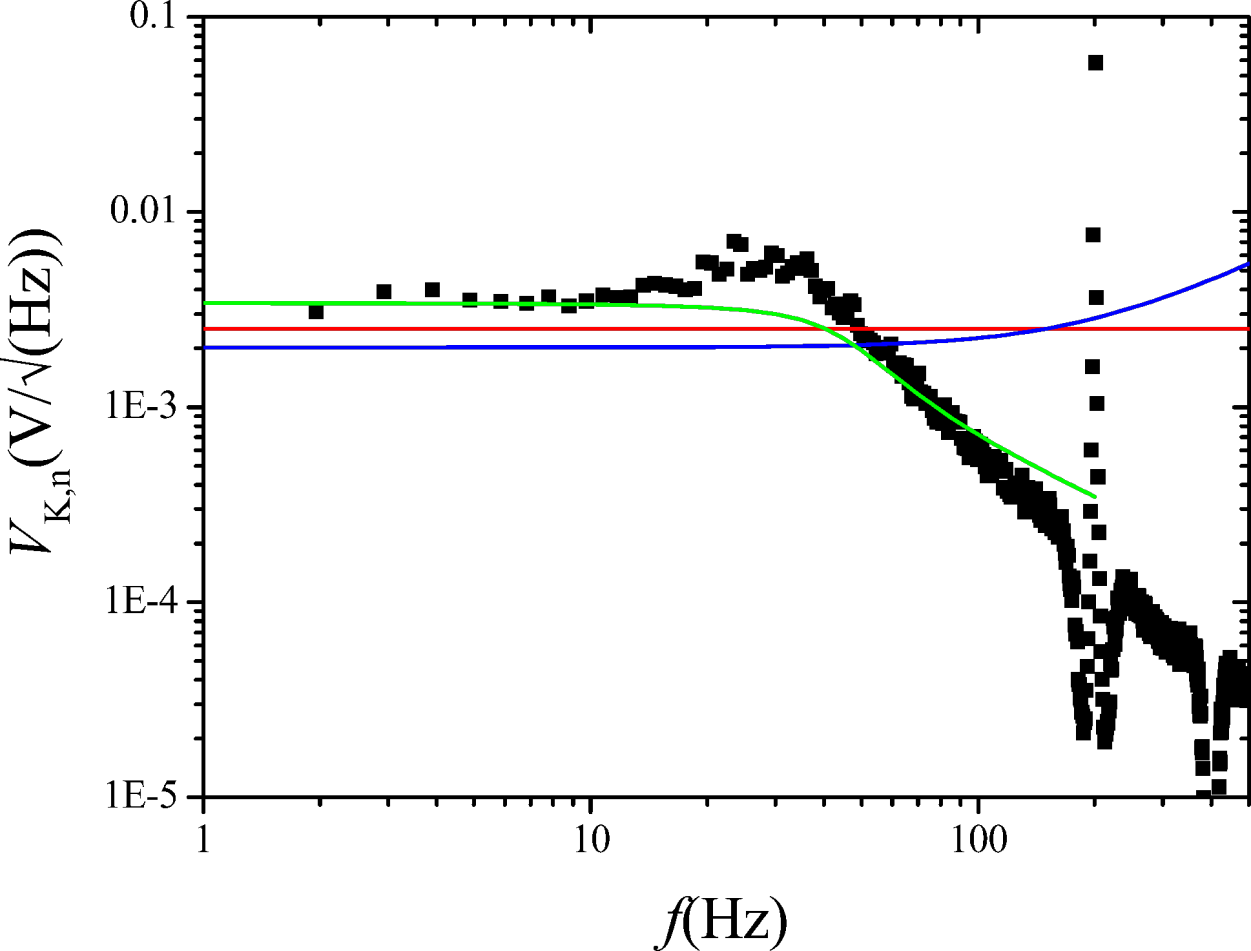
Measured (black squares) and computed (green line) Kelvin closed loop noise PSD of the setup of Figure 10. Also shown is the decomposition into thermal (red, constant) and sensor (blue, rising above 200 Hz) noise, calculated by using the approximate noise gain.

The results are shown in Figure 13 and Figure 14 respectively. The fits have been obtained by using as output filtering of the Kelvin lock-in, *H*_lockin_(*f*_pert_), a second order function with cutoff at 60 Hz. This cannot be set manually in our case and is thought to be directly coupled to *f*_mod_ = 200 Hz.

The observation that the closed loop Kelvin response measured with engaged distance control is in agreement with the modeling based on the PLL response determined with retracted tip, supports the assumption that the gain and distance control loops with a setpoint of Δ*f* = −5 Hz do not interfere with the Kelvin control loop by modifying the forward gain of the PLL. This situation corresponds to a weak surface interaction in the sense of [7].

The green curve of Figure 14 shows the numerically computed noise according to the exact expression of Equation 30 and Equation 31 and the demodulated noise of Equation 27. The red and the blue curves are the decomposed thermal and sensor noise calculated from the approximate noise gain of Equation 31 and the demodulated noise of Equation 27. Both computations do not reflect the little harmonic overshoot of the spectrum at around 25 Hz. Again, the approximation by using noise gain is accurate only up to the roll-off of the closed loop response. The sensor noise component of *V*_K,n_ is relatively constant in contrast to the rising sensor noise at the PLL output because it is the average between the satellites at *f*_mod_ ± *f*_pert_.

## Discussion

Up to here, a typical laboratory setup has been treated in order to validate numerical and analytical treatment of noise propagation. Here, the cutoff frequency of the Kelvin loop had been set to 30 Hz by the choice of the feedback parameters as shown in Figure 12. With a Kelvin noise PSD of around 4 mV/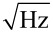, the total noise is expected to be around 22 mV. The bandwidth is an arbitrary choice and is limited by the acceptable noise level. The PLL bandwidth could indeed be set to a value in the kHz range, allowing to increase the AC modulation frequency and bandwidth of both distance and Kelvin control loops. In the following, the constraints with respect to a maximum bandwidth to noise performance shall be addressed.

### Choice of *f*_mod_ with respect to bandwidth BW

**Figure 15:**
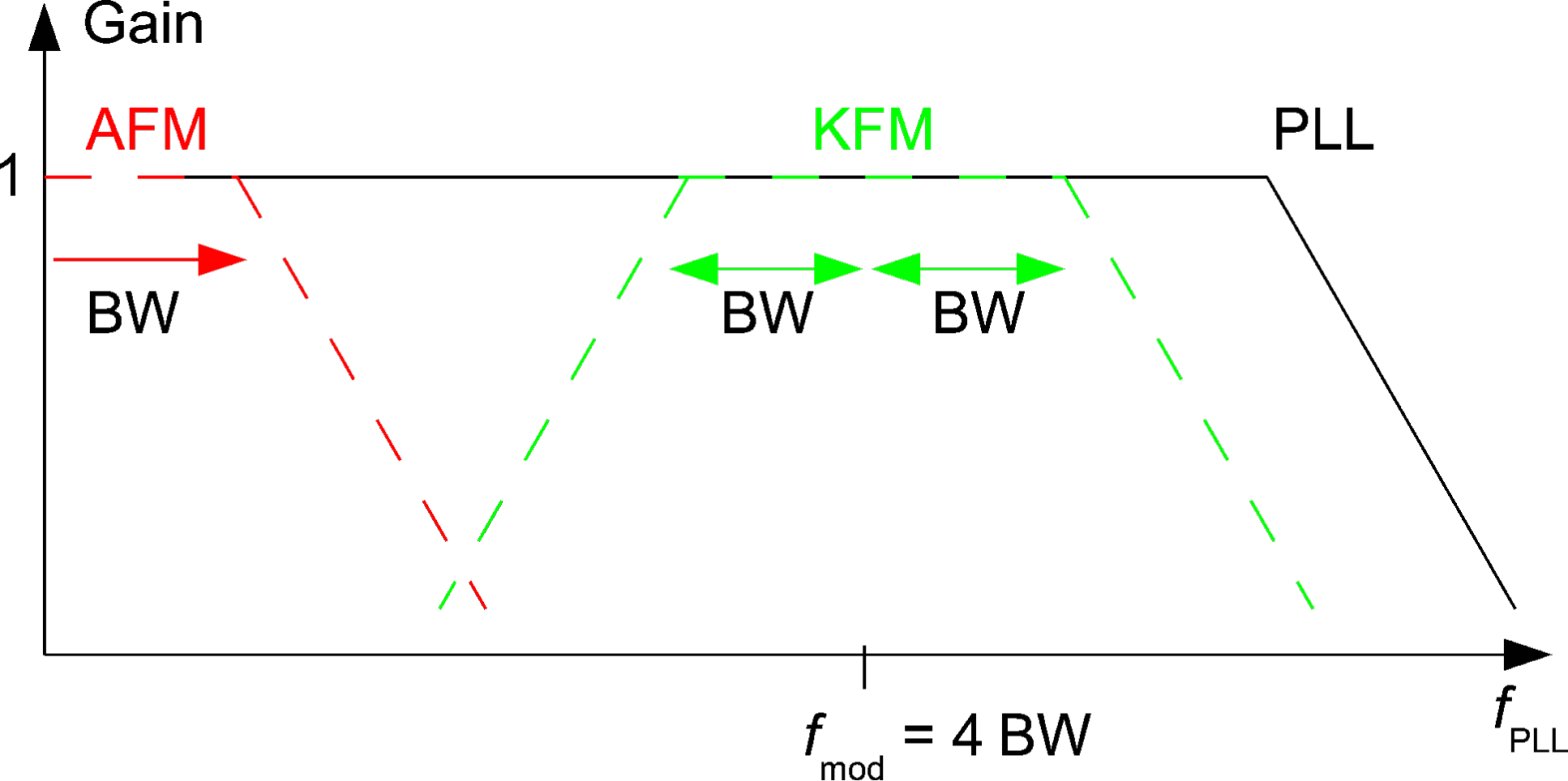
Design rule for cutoff and modulation frequencies in FM-KFM: gain of the PLL controller (continuous black), gain of the distance controller (red dashed), and operating range of the Kelvin loop in terms of PLL frequency (green dashed).

A design rule for the choice of the different frequencies is given in Figure 15: the black (solid) curve schematically represents the gain of the PLL controller. The red (dashed) curve is the gain of the distance controller. The green (dotted) curve is the range in terms of PLL frequency occupied by the Kelvin loop, consisting of two satellites of the Kelvin response around the AC modulation frequency. It is reasonable to plan the bandwidth of the distance control loop to be equal to the one of the Kelvin controller, *f*_c,AFM_ = *f*_c,KFM_ = BW, since usually both images are sampled at the same rate because it is a one pass technique and the Kelvin image is typically acquired with the same resolution as the topography image. If the modulation frequency is chosen to be *f*_mod_ ≈ 4*f*_c,AFM_, then the Kelvin loop is using the PLL in a frequency range up to *f*_mod_ + *f*_c,KFM_ = 5 BW, which should be at a value such that the total noise remains acceptable (see section “Kelvin voltage noise PSD”), and on the other hand, the overlap and hence crosstalk between topography and KFM image is small since the roll-off of the distance controller at 1 BW and of the lower PLL frequency satellite of the Kelvin controller at *f*_mod_ − *f*_c,KFM_ = 3 BW are 2 BW apart. The cutoff frequency of the PLL, the AC frequency and the bandwidth of the Kelvin loop can be set to much higher values as discussed in the section “Absolute frequency limits irrespective of noise”. However the effective noise PSD of the PLL is composed of thermal and detector noise, shown in Figure 5, Figure 7 and Figure 9 and by Equation 15 and Equation 19. Care has to be taken that total noise, i.e., the integral of the noise PSD over the operating range, remains acceptable.

The 2 BW gap between the roll-off frequencies, together with the finding that both closed loop responses are second order systems, ensures that a CPD represented by *V*_pert_ varying at *f*_c_ = BW causes a response of the distance controller at 3*f*_c_ at −24 dB below its response to a static CPD. According to Equation 24, an AC CPD represented by *V*_pert_, as well as the AC voltage *V*_mod_, also both introduce a static term. The crosstalk onto the distance controller of *V*_pert_ oscillating at *f*_c_ introduces an oscillation of *z* at 3*f*_c_:

[32]
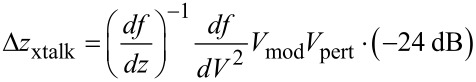


The first two factors are

[33]
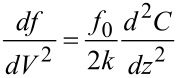


and

[34]
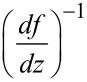


which is highly non-linear and dependent on the Δ*f* setpoint. It is sufficient to know the product. This can be determined from the static term:

[35]
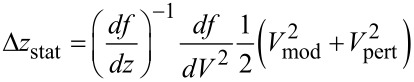


It is appropriate to choose a Δ*f* setpoint such that the tip does not retract considerably in response to the applied value of *V*_mod_. Equation 24 contains terms that cause a static tip retraction, and a dynamic tip movement at 3*f*_c_ and 5*f*_c_ due to mixed terms, and at 2*f*_c_ and 8*f*_c_ due to squared terms. If amplitudes are equal, *V*_mod_*V*_pert_ = 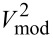 = 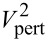, then the dynamic tip retraction at 3*f*_c_ is −24 dB below the sum of the constant terms, or 6%. The constant tip retraction can be thought to be less troublesome because it introduces only an offset in the topography image while the retraction from varying surface potential introduces a real artifact. Nevertheless, it is favorable to minimize the tip–sample distance since it deteriorates the lateral resolution. Setpoint Δ*f* and *V*_mod_ should be chosen such that the topography feedback is still dominated by van-der-Waals interaction. However, the tip–sample separation cannot be made infinitely small by hardening the topography feedback because of the snap-to-contact phenomenon. The ultimate limit is discussed below, and constraints between tip–sample separation, oscillation amplitude, and *V*_mod_ enter into a probe merit factor.

The electrostatic force terms of Equation 24 at 5*f*_c_ and at 8*f*_c_ are even further apart from the distance controller cutoff frequency. The term at 2*f*_c_ does introduce some response of the distance controller, but this has a negligible effect on the Kelvin controller that demodulates at 4*f*_c_. Vice versa, the crosstalk of a topography varying at *f*_c_ onto the *V_K_* voltage is a variation at 3*f*_c_ damped by 24 dB:

[36]
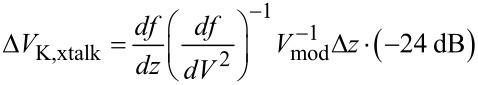


### Kelvin voltage noise PSD

The thermal noise PSD of the PLL frequency noise Equation 19 is constant and hence invariant under the frequency translation, yielding as Kelvin noise PSD, by dividing through the Kelvin gain Equation 26,

[37]
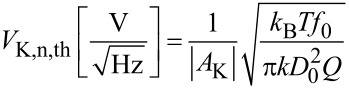


indicated as red curve in Figure 14. The integrated noise is

[38]
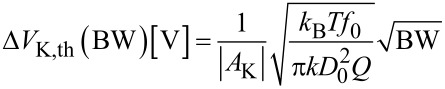


while the sensor noise of Equation 19 contributes to the Kelvin noise PSD

[39]
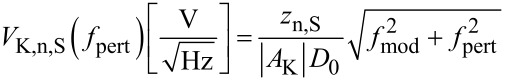


(blue curve of Figure 14). The integrated noise due to sensor noise, still with the condition that *f*_mod_ = 4 BW, is

[40]
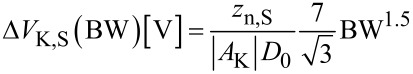


The following treatment supposes that one of the noise sources is dominant and hence the total integrated noise Δ*V*_K_ is either equal to Δ*V*_K,th_ or to Δ*V*_K,S_.

### Merit factor and design optimization

We define the merit factor as

[41]
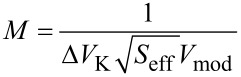


To obtain a merit factor, it is necessary to divide the reciprocal of the integrated noise Δ*V*_K_ by the root of the effective probed surface *S*_eff_. It is obvious and a basic rule of statistics that a potential measurement on a *n* times bigger surface made in the same time with the same state of the art of measurement apparatus has a fluctuation of 1/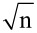 times the one on a simple surface.

We also divide by the AC voltage *V*_mod_ since for otherwise identical conditions, the Kelvin forward gain *A*_K_ given by Equation 26 is proportional to it but at the same time this voltage has the effect of introducing an error on semiconductors by asymmetric band bending. The subject has been addressed by several authors [17–20]. If KFM is performed on a semiconductor, the AC bias applied to the tip causes a response of the underlying semiconductor that alternates between majority-carrier depletion and accumulation. The tip–substrate junction can be thought of as a capacitive voltage divider formed by the tip–substrate capacitance and the Mott–Schottky capacitance. We expect this description to be valid over a wide frequency. The competing process of inversion-layer buildup has a time constant that is typically on the order of seconds to minutes for industrial grade semiconductor and hence negligible even in FM-KFM. If charge capture and emission by defect states is involved, it is imaginable that time constants are such that frequency dependence or non-linearity can play a role. Due to the lack of detailed knowledge, we justify dividing the merit factor by *V*_mod_.

The integrated noise Δ*V*_K_ is dominated by thermal or detector noise depending on bandwidth and temperature. We define as crossover temperature *T*_cross_ the temperature above which at a given bandwidth, the integrated thermal noise of Equation 38 exceeds the integrated sensor noise Equation 40, while the design rule is respected:

[42]
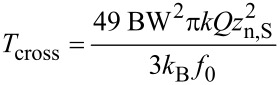


**Figure 16:**
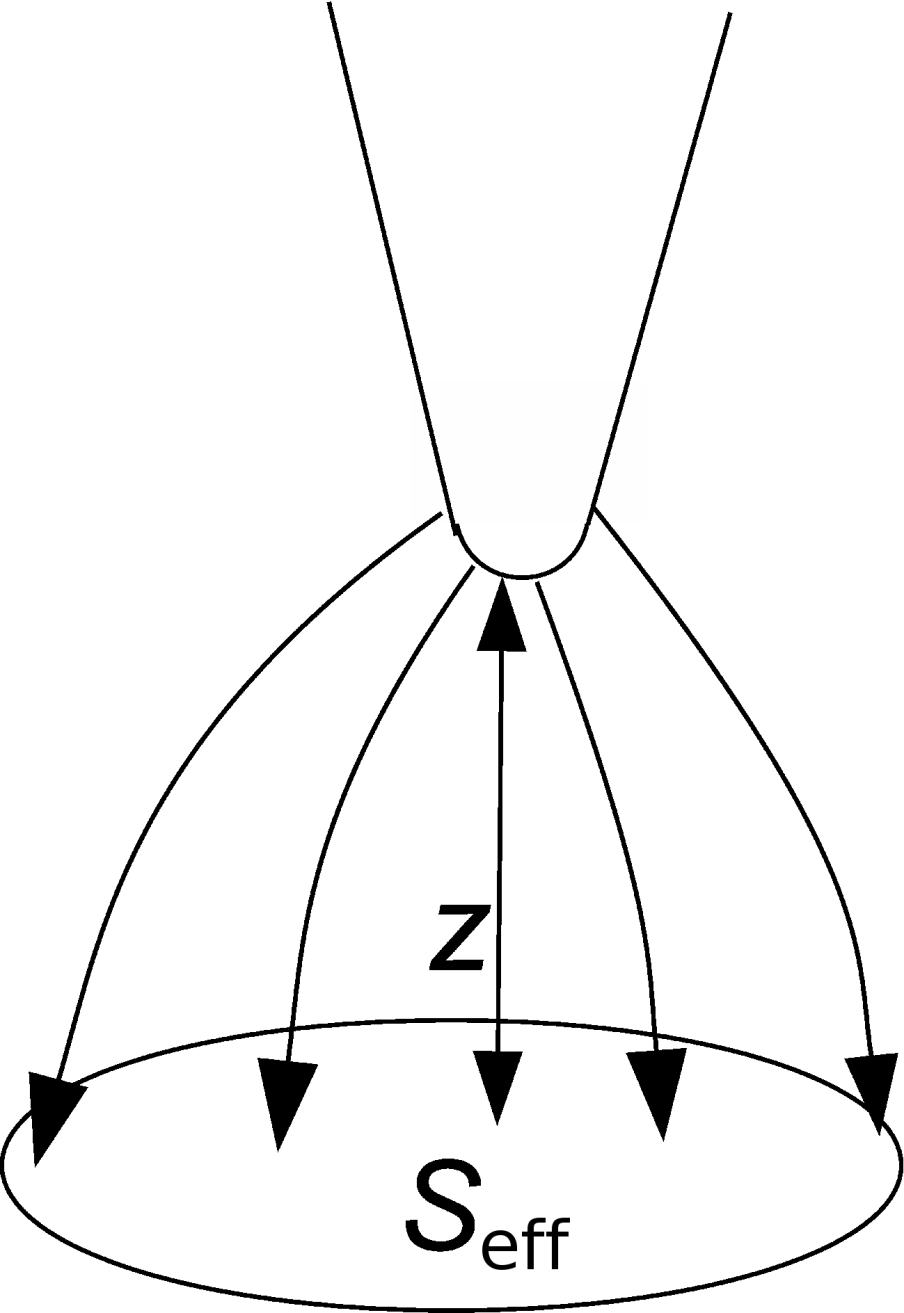
Effective probed surface *S*_eff_ depending on tip–sample separation *z*.

Regarding the effective probed surface *S*_eff_, its absolute value is not known, but the relation between tip–sample distance and probed surface, as illustrated in Figure 16, is described by a power law derived from the second derivative of the capacitance [21]. Here we make the approximation that the probe oscillates with a small amplitude *D*_0_ around a larger average probe distance *z*.

[43]
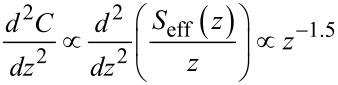


Hence

[44]
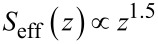


For thermal noise domination, Equation 38, the merit factor is

[45]
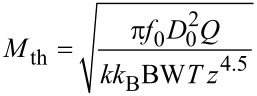


If it is assumed that the maximum oscillation amplitude *D*_0_ cannot exceed a certain fraction of *z* and hence is proportional to it, it reduces to

[46]
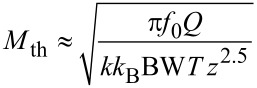


Furthermore, a relation has to be respected between minimum tip–sample distance *z* and spring constant *k* to avoid snap to contact.

**Figure 17:**
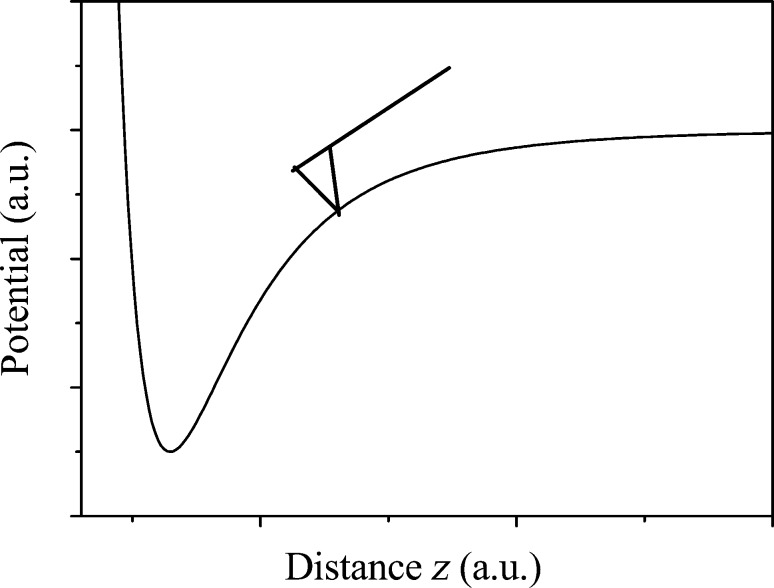
Probe in the attractive part of the Van-der-Waals interaction.

Figure 17 shows the tip in the attractive part of the van-der-Waals interaction. The force gradient in this field must not exceed the spring constant to avoid snap to contact. We take the attractive range of a Lennard-Jones type of potential

[47]
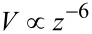


The force gradient is proportional to the second derivative:

[48]
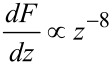


To avoid snap to contact, the force gradient must be smaller than the cantilever stiffness

[49]
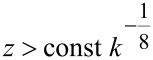


And hence Equation 46 reduces to

[50]
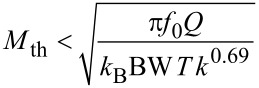


For comparison, a widely used merit factor for MEMS resonators is

[51]
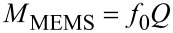


and the one of minimum force detection is

[52]
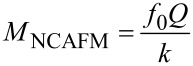


This result, i.e., the maximization of *f*_0_*Q*/*k*^0.69^ is positioned between the usual MEMS benchmark *f*_0_*Q* and a merit factor *f*_0_*Q*/*k* found by Albrecht [3] for the minimum detectable force by noncontact AFM.

If the noise PSD is dominated by detector noise, Equation 40, then we obtain a merit factor *M*_S_ instead of Equation 45:

[53]
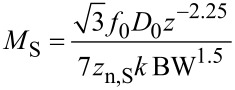


similarly as above, *D*_0_ is a fraction of *z* and hence

[54]
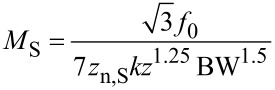


Using Equation 49 for the relation between *z* and *k* yields

[55]
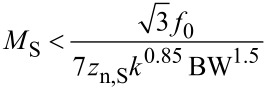


Unsurprisingly, for the case of dominating sensor noise, maximization of the merit factor requires minimizing the sensor noise. Both merit factors, Equation 50 and Equation 55, suggest downsizing both the probe spring constant and mass. If one considers *f*_0_ = 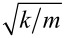, the exponents of *k* higher than 1/2 in the denominator yield increasing merit factors for decreasing stiffness. Both merit factors cannot be increased infinitely because downsizing the probe beyond a certain limit will decrease the *Q*-factor and increase sensor noise.

A table of merit factors for thermally dominated noise, sensor dominated noise and crossover criteria is given in Table 1. The table lists probe parameters, followed by a crossover criterion, Equation 42, the crossover temperature for a bandwidth of 50 Hz, the merit factor for dominant thermal noise according to Equation 50, and the merit factor for dominant detector noise according to Equation 55. For the stiffness of the Kolibri sensor, we use 1 MN/m, about the double of what is given in the documents from Specs [22]. The 540 kN/m is the spring constant of the entire needle which is suspended in the middle. In SPM operation, the two prongs are moving oppositely and the suspension remains stationary. Therefore, for comparison with the other probes, the effective stiffness of twice the given value has to be used. The use of the table for comparison of probe performance consists in first determining the crossover temperature as function of the desired bandwidth by multiplying the value *T*_cross_/BW^2^ with BW^2^. If the working temperature is below the obtained crossover temperature, the merit factor *M*_S_ applies and is obtained by dividing the value *M*_S_·BW^3/2^ through BW^3/2^. If the working temperature is above the crossover temperature, the merit factor *M*_th_ applies and is obtained by dividing *M*_th_·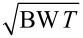 by 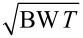. The dominating merit factor among *M*_S_ and *M*_th_ is the one with the lowest value, due to its definition containing the reciprocal of *V*_K,n_, according to Equation 41. The performance of probes with thermally dominated noise can be compared directly to others with dominating sensor noise.

**Table 1:** Key values, crossover criteria, and merit factors for different probes.

	Cantilever (Nanosensors)	qPlus Sensor (Giessibl, 2011 [9])	Kolibri Length Extension Resonator (Specs [22])	IEMN Disk Resonator (Algre 2012 [23])

	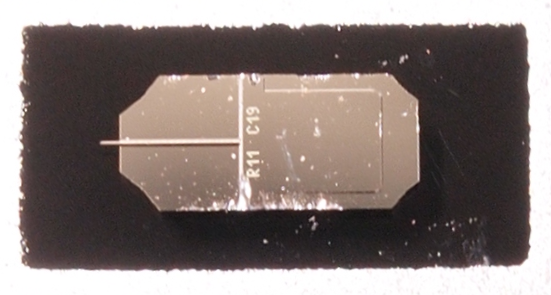	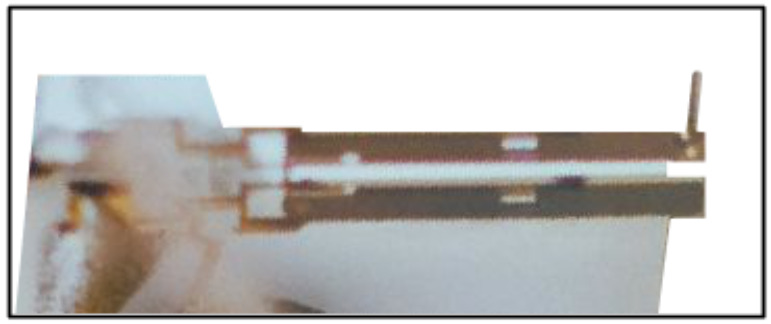	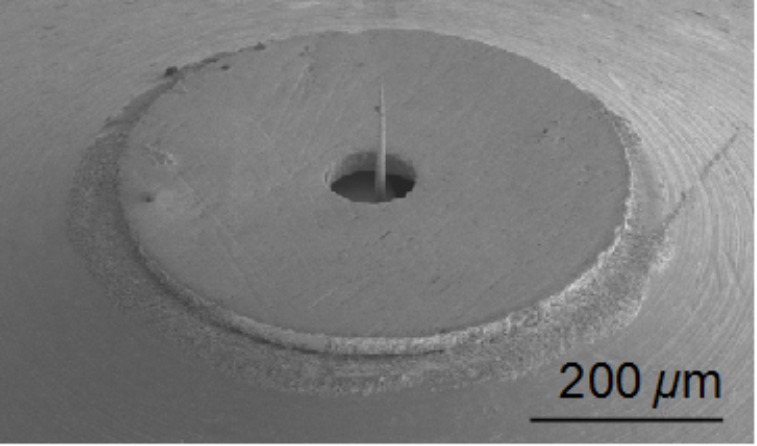	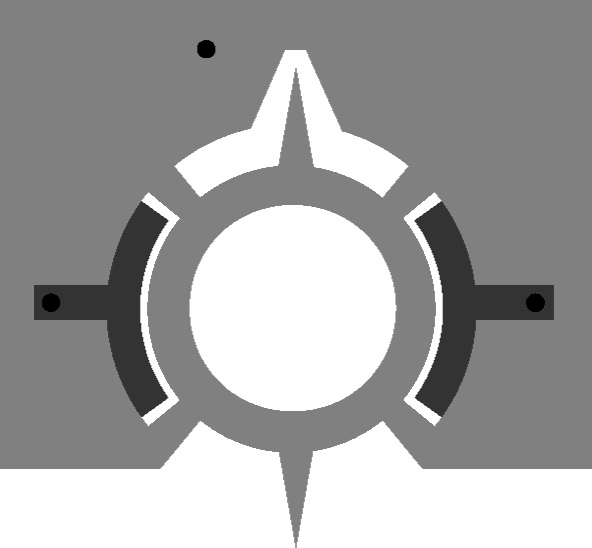

*f*_0_ (Hz)	61836	30k	1M	1.45M
Q	22k8	200k	14k	1k6
*k* (N/m)	1.2	1k8	1M	135k
*Z*_n,S_ (m/ 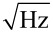 )	2e-13	6.2e-14	1e-15	1e-12
*T*_cross_/BW^2^ (K/Hz^2^)	6.81e-2	177.5	5.38e-2	594
Crossover Temp (K) at BW = 50 Hz	170	444k	135	1.48M
*M*_th_· 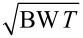	1.71e16	2.83e15	4.89e14	3.97e14
*M*_s_·BW^3/2^	6.75e16	2.05e14	1.97e15	1.56e13

The table shows that cryogenic cooling is useful only for reducing the thermal excitation of the Kolibri sensor and to some extent of cantilevers, whereas the qPlus and disk resonator have dominant detector noise at all achievable temperatures, recognizable by crossover temperatures in the kilo- or Mega-Kelvin range. (Detector noise was assumed temperature independent). The best FM-KFM performance is expected from standard cantilevers. It can be expected that these probes in combination with interferometric detection might benefit from cooling to temperatures even below liquid helium. Despite significant performance differences, the existence of all compared probe types seems to be justified. For instance, some environments require a need for electrical rather than optical deflection detection, and the performance criteria for topography imaging differ largely from the FM-KFM merit factor, due to the highly non-linear probe sample interaction that motivates a wide range of cantilever stiffness and oscillation amplitudes.

## Absolute frequency limits irrespective of noise

The example treated here seems to have rather low performance compared to, e.g., video-rate SPM setups that claim to image biological processes in real-time (however in topography mode only). We emphasize that the choice of our bandwidth is our personal preference of making the compromise between bandwidth and noise. As stated above, the 30 Hz bandwidth leads to 22 mV signal fluctuation. Since the sampling circuitry has an anti-aliasing filter that cuts above half the sampling rate and it is not justified to smooth the image by slow response of the Kelvin and topography loop responses, we can acquire at 50 pixel per second, meaning that a line with 256 pixels is scanned back and forth in 10 seconds and an image at 256 × 256 resolution takes 45 minutes. We are used to acquire images with higher resolution over night. Since the output noise has been tracked down to thermal excitation and displacement detector noise, said compromise has universal validity. We also mention here that the exchange of the light source in the optical beam deflection sensor has already decreased the detector noise by an order of magnitude with respect to the original value, and that otherwise for the same choice of bandwidth, the detector noise would be dominating and the Kelvin signal fluctuation would be a multiple. In this short paragraph we address the question to what extent speed can be increased at the expense of noise and when other limitations apply.

- PLL bandwidth: for phase locked loops, the terms capture range and lock range denominate the frequency range in which the PLL can lock on to an incoming signal and maintain the phase lock. It is given as percentage of center frequency, depends on the degree of sophistication of the circuit (phase detection, filters) and is above 10 percent even for primitive monolithic circuits that use edge detection and simple filters such as the NE567 PLL tone decoder. The capture range is always below the lock range. The given percentage is the frequency shift of the frequency modulated signal, which is a function of both the excursion frequency and the modulation frequency. Without entering PLL theory in detail, we can say that a PLL bandwidth of 10 percent of the center frequency is realistic and it has been experimentally confirmed that our PLL bandwidth can be set to 5 kHz.
- AC frequency *f*_mod_ and PLL bandwidth BW_PLL_: these frequencies have to be chosen such that *f*_mod_ + BW ≤ BW_PLL_. Together with the design rule, for our example, we would obtain *f*_mod_ = 4 kHz and BW = 1 kHz.
- Distance control: this component is probably the most limiting. In our setup where the sample is mounted on a 3 axis piezo scanner, the bandwidth is limited to a value between 100 and 200 Hz.

Consequently, if we had set the PLL bandwidth BW_PLL_ = 5 kHz, the AC frequency *f*_mod_ = 4 kHz and the bandwidth of the Kelvin controller BW = 1 kHz, according to Figure 9 the detector noise would be dominating and due to the power law with exponent 3/2 of Equation 40, we would expect Kelvin voltage fluctuation in the volt range. Furthermore, the distance control would not be able to keep up with the Kelvin loop.

## Perspective on the ultimate probe and detector

It is obvious from the two merit factors that a reduction of both thermal and detector noise at the same time is difficult. If thermal excitation is dominant and the effort aims at reducing it, the frequency range where it dominates becomes smaller, as can be seen in Figure 9. Mass and spring constant cannot be reduced infinitely without reducing the *Q*-factor. Furthermore, increasing the merit factor in the thermally dominated case is a simple downsizing of the detector, and with the same type of sensor, would increase the sensor noise or decrease the *Q*-factor of the oscillator by sensor back-action (e.g., radiation pressure). Similarly, all attempts of improving the detector have a trend to increase invasiveness and to reduce the *Q*-factor. As long as one type of noise is clearly dominant, the remedy is to maximize the respective merit factor, keeping in mind the above dependencies. Present state of the art for measuring the excursion of harmonic oscillators consists in optical interferometry [24] or single electron transistors [25] used as position probe coupled to oscillators, combined with cooling of the resonator to cryogenic temperatures, possibly using laser cooling. These works aim at the Heisenberg limit and are not specific to scanning probe microscopy. Practical SPM systems seem to be still further away from the ultimate limit.

## Conclusion

The dynamic behavior of an FM-KFM has been measured and modeled for a system with characteristics typically obtained in ultrahigh vacuum implementations. It has been shown that in a PLL based setup, the two main noise sources, thermal excitation and detector noise, transform into frequency noise exactly the same way as in a free-running oscillator, and that the PLL components do not contribute considerable noise, meaning that the main noise sources are sufficient to derive Kelvin voltage noise. Feedback parameters for PLL and Kelvin loop have been set for a stable behavior and been used for the numerical modeling of the noise propagation, yielding output noise spectra in agreement with the measurements. The choice of the AC modulation frequency to be four times the intended bandwidth has been proposed and justified as design rule. Based on the acquired knowledge, the KFM performance has been modeled for three other well-known AFM probes. A crossover criteria allows one to determine for each probe, depending on temperature, detector noise PSD, bandwidth and probe parameters, whether Kelvin output noise is dominated by thermal probe excitation or by detector noise. Depending on the regime, one of two merit factors apply to obtain the overall noise performance from instrument parameters, to suggest improvements and to allow for a comparison of different probes. Limitations to the optimization remain due to unresolved interdependent parameters, the trend of entering a thermally limited regime when improvement is made to detector noise and vice versa, and deteriorating one noise source when improving the other, ultimately merging into the uncertainty relation governing that a system cannot be measured without changing it by whatever kind of sensor back-action.

## Experimental

The KFM is based on an Omicron ultrahigh vacuum variable temperature atomic force microscope (UHV-VT-AFM). It is operated by a Nanonis scanning probe microscopy (SPM) controller entirely based on digital signal processing (DSP). The probe that was used in these experiments is a platinum-iridium coated Nanosensors Point Probe Plus EFM tip with a spring constant between 1 and 3 N/m. Its resonance frequency *f*_0_ = 61.835 kHz and the *Q*-factor *Q* = 22800 were determined in vacuum by recording a resonance curve with the built in function of the Nanonis controller. The optical beam deflection detection uses a 20 mW Superluminescent (TM) light emitting diode that was operated at an intensity of 7 mW. About 0.5 mW intensity is received by the photodiode, which was estimated from its known current–intensity characteristics. To compensate the increased intensity of the light source, the gain of the transimpedance amplifier was reduced accordingly to avoid output voltage saturation. The sample is a gold coated silicon substrate (Omicron test sample). KFM measurements are performed while distance control is enabled.
